# Intracellular enzymatic reducing systems control receptor tyrosine kinase signaling via PTP1B

**DOI:** 10.1126/sciadv.adv5362

**Published:** 2026-04-10

**Authors:** Lucia Coppo, Wenchao Zhao, Qing Cheng, Axel Tobias Scholz, Elias S. J. Arnér, Markus Dagnell

**Affiliations:** ^1^ Division of Biochemistry, Department of Medical Biochemistry and Biophysics, Karolinska Institutet, SE-171 77 Stockholm, Sweden.; ^2^ Department of Selenoprotein Research and National Tumor Biology Laboratory, National Institute of Oncology, Budapest, Hungary.

## Abstract

Protein tyrosine phosphatases (PTPs) counteract receptor tyrosine kinase (RTK) signaling. Inhibition of PTPs by oxidation can be reversed by cytosolic thioredoxin (TXN), but less is known about regulation of PTPs by glutathione (GSH)–driven glutaredoxins (GLRXs). Here, we thus assessed GLRX1, GLRX2, and/or TXN1 in regulation of CO_2_/bicarbonate- and H_2_O_2_-mediated oxidation of the physiologically important PTP1B. GLRXs and TXN1 synergistically maintained PTP1B activity, and modulating cellular levels of either GLRX1, GLRX2, or TXN1 gave strong effects on phosphorylation cascades triggered by epidermal growth factor (EGF) or platelet-derived growth factor (PDGF). Furthermore, transient intracellular interactions of PTP1B with GLRX1, GLRX2, and TXN1 were discovered within minutes after stimuli with either PDGF or EGF, coinciding with control of the corresponding RTK-driven phosphorylation cascades. We conclude that TXN1 and GLRXs are key regulators of PTP1B activity and thus control cellular responses to RTK stimulation.

## Teaser

A cross-talk among the antioxidant GLRXs and TXN1 enzyme systems directly regulates PTP1B activity during growth-factor signaling.

## INTRODUCTION

Signaling via growth factors such as epidermal growth factor (EGF) or platelet-derived growth factor (PDGF) determines cellular decisions of differentiation, migration, and proliferation through activation of receptor tyrosine kinases (RTKs) (*1*). Ligand binding to an RTK induces dimerization and transphosphorylation of the intracellular part of the RTK dimer, triggering signaling through downstream protein phosphorylation cascades. This is counteracted by a family of protein tyrosine phosphatases (PTPs), thus being key enzymes regulating RTK signaling. Imbalances in RTK or PTP activities can contribute to disease progression in pathologies such as cancer and diabetes (*1*, *2*). To enable phosphorylation events triggered by RTKs, PTPs are temporarily inhibited via reversible oxidation of their conserved active site Cys residue (*3*, *4*). This active site Cys of PTP1B with a low p*K*_a_ (where *K*_a_ is the acid dissociation constant; 5.5) is in the thiolate form at physiological pH, thereby facilitating oxidation to take place (*1*, *3*, *5*, *6*). Notably, binding of growth factors, with signaling through RTKs, including EGF and PDGF, induces a receptor-dependent transient increase in H_2_O_2_ production through activation of membrane localized NADPH (reduced form of nicotinamide adenine dinucleotide phosphate) oxidases (NOXs) (*7*, *8*). Upon activation, the NOXs produce superoxide that is converted into H_2_O_2_, facilitated by extracellular superoxide dismutase. H_2_O_2_ is then brought into the cytosol by aquaporins (peroxiporins) (*9*, *10*) where it may react with cellular CO_2_/bicarbonate to form peroxymonocarbonate (*11*, *12*) that potently oxidizes and inactivates PTPs (*13*). The mechanism of peroxymonocarbonate formation is not fully understood but likely involves facilitation by the PTPs themselves. We have recently shown the importance of cellular bicarbonate during RTK signaling, where the presence of bicarbonate determined the degree of PTP1B oxidation both in vitro and in cells, and was required for EGF signaling (*14*). Hence, oxidation of PTPs is an integral event in RTK signaling. Reversible oxidation of the active site Cys of PTP1B can lead to the formation of its sulfenic acid (-SOH) derivative (*15*, *16*) that, in turn, can form an intramolecular cyclization with an amide nitrogen into a sulfenylamide (*17*–*19*). Other reversibly oxidized forms of PTP1B include glutathionylation (*20*, *21*), persulfidation (*22*, *23*), and nitrosylation (*24*). These reversible modifications may protect PTP1B from irreversible oxidation. Reactivation of reversibly oxidized PTP1B by reduced glutathione (GSH), in a two-step mechanism, was characterized by the Gates laboratory (*25*), and PTP1B was also shown to be glutathionylated during EGF receptor (EGFR) signaling (*26*). Another regulator of PTP oxidation is the scaffold protein 14-3-3ζ that was shown to interact with and stabilize PTP1B in its sulfenylamide form (*27*). Several different PTPs including PTP1B were shown to be subject to reversible oxidation in A431 cells, as triggered by endogenously produced reactive oxygen species while hampered by inhibition of NOXs, which was shown using an activity-based “modified in-gel PTP assay” (*28*).

Redox regulation of PTP activity and thereby regulation of RTK signaling should depend upon the combined effects of oxidation through the action of NOXs, and the reduction of the oxidized active site Cys residue. These events are in turn likely to be modulated by the activities of the two main cytosolic antioxidant systems, i.e., the thioredoxin (TXN) system including TXN1 (*29*) or GLRXs, more specifically GLRX1 and GLRX2 (*30*). The highly abundant TXN1- and GLRX-dependent peroxiredoxins (PRDX) are very efficient H_2_O_2_ peroxidases and thereby control the cellular levels of oxidants (*31*–*33*) and indirectly modulate PTP activity and RTK signaling (*14*, *34*). GLRXs catalyze the reduction of glutathionylated proteins, leading to the formation of GSH disulfide (GSSG), with reduction of GSSG back to GSH catalyzed by GSH reductase (GSR) (*30*). Previous studies also indicate a regulatory role of the GSH system on PTP activity, identifying a differential sensitivity of TXN1 and GLRXs toward oxidized PTPs (*15*). Direct glutathionylation of PTP1B was also shown in vitro with reactivation by GLRX1 (*21*). Overexpression of GLRX1 in H9c2 cells was also found to regulate PDGF-dependent phosphorylation and proliferation by controlling the activity of low–molecular weight PTP (LMWPTP) (*35*).

Here, we aimed to further investigate the modulation of PTP1B oxidation by GLRXs and TXN1. We discovered a major cross-talk among the two antioxidant systems, including a transient intracellular protein-protein interaction between PTP1B and GLRX1 as well as GLRX2, and between PTP1B and TXN1, triggered by PDGF or EGF growth-factor signaling. These interactions were localized to the perinuclear area, indicating that reduction of PTP1B takes place in vicinity of the endoplasmic reticulum. This work is aiming to better characterize the temporal regulation of cellular antioxidant systems controlling RTK signaling through PTP1B and conduct a side-by-side comparison of PTP1B activity support by GLRX1, GLRX2, and TXN1. The results reveal how both GLRXs and TXN1 are key regulators of RTK-coupled phosphorylation cascades.

## RESULTS

### EGF-induced protein tyrosine phosphorylation in A431 cells is decreased by overexpression of GLRX1 or GLRX2

**Fig. 1.**
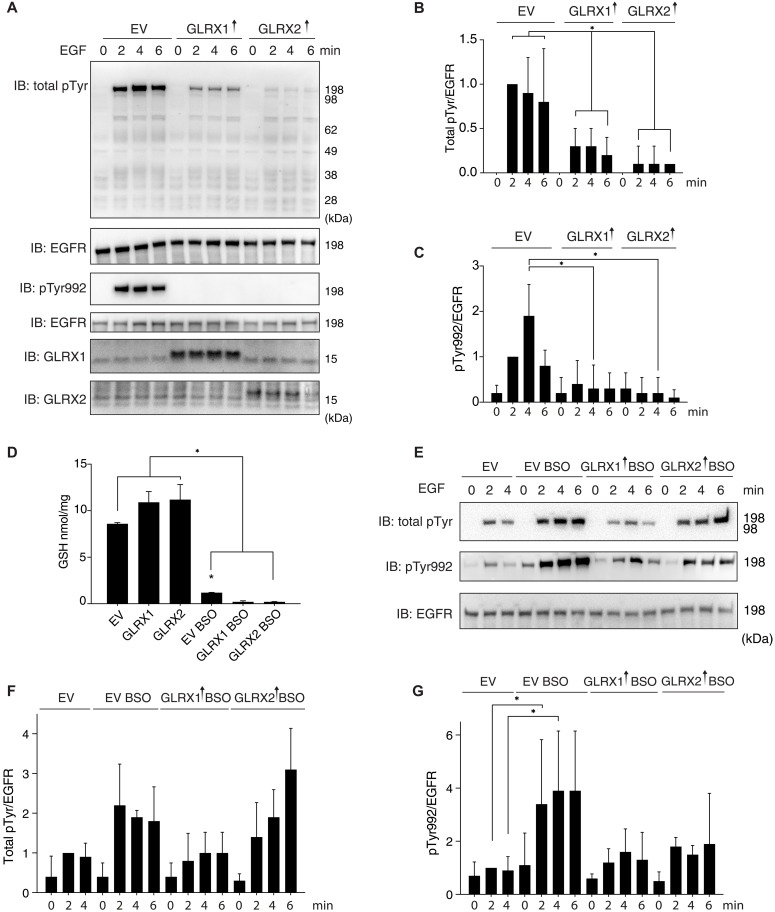
Overexpression of GLRXs leads to a decrease in tyrosine phosphorylation in A431 cells stimulated with EGF. (**A**) A431 cells overexpressing GLRX1 or GLRX2, indicated with upward arrows, were serum starved before stimulation with EGF (100 ng/ml) for 0-2-4-6 min. Lysates were analyzed with immunoblots (IB) for total EGFR, for total phosphotyrosine (total pTyr), for the preferred site for PTP1B-mediated dephosphorylation (pY992), and for GLRX1 or GLRX2 expression levels, as indicated. (**B** and **C**) Quantifications using densitometry of total pTyr and pTyr992, respectively. The signals were normalized for the intensity of the total expression of EGFR and compared with signals in controls. (**D**) GSH levels were determined in A431 cells with or without overnight treatment with 125 μM buthionine sulfoximine (BSO), as indicated. EGF stimulation and GLRX1 or GLRX2 overexpression as in (A). (**E** to **G**) Immunoblots and quantification of total pTyr and pTyr992 in the cells analyzed in (D). The signals were normalized for the intensity of the total expression EGFR. EV, cells transfected with control vector; GLRX1↑ and GLRX2↑, cells transfected for overexpression of GLRX1 or GLRX2, respectively. Bar graphs are determinations from immunoblots of three separate cell experiments [*n* = 3; means ± SE (error bars); *P < 0.05]. Data were analyzed using one-way analysis of variance followed by Bonferroni post hoc tests for multiple comparisons with GraphPad Prism.

To analyze the possible importance of GLRXs during EGF-triggered signaling, we first overexpressed either GLRX1 or GLRX2 in human epidermoid carcinoma A431 cells, expressing high levels of EGFR (*36*). For this, serum starved cells were stimulated with EGF ligand (100 ng/ml) for 2, 4, and 6 min before harvest. Analyses of total protein phospho-tyrosine levels revealed that overexpression of either GLRX1 or GLRX2 significantly decreased the extent of EGF-induced Tyr phosphorylation (Fig. 1, A and B). In particular, the specific phosphorylation site Tyr^992^ (pY992) of the EGFR, known to be a preferred site for PTP1B-mediated dephosphorylation (*37*), showed a marked decrease in phosphorylation, thus indirectly indicating an increase in PTP1B activity (Fig. 1, A and C).

Because oxidized GLRXs are recycled back to their reduced form using GSH, we next explored the effects of GSH depletion on the Tyr phosphorylation pattern as triggered by EGF. To deplete the cellular GSH pool, cells were pretreated with buthionine sulfoximine (BSO), an inhibitor of ɣ-glutamyl-cysteine synthetase that is a key enzyme for GSH synthesis. Analysis of GSH levels confirmed the depletion of GSH (Fig. 1D). When cells were stimulated with EGF under these conditions, a strong time-dependent increase in both total and PTP1B site–specific phosphorylation was found, which could partially be reverted by overexpression of GLRX1 and, to a lesser extent, by GLRX2 (Fig. 1, E to G). With GLRXs typically depending on reduced GSH for their functions, only one cycle of reduction would be likely in the absence of GSH unless the GLRXs would also be reduced in some alternative non–GSH-dependent manner. Irrespective of the exact molecular mechanisms of action, these results, however, showed that GLRX overexpression and GSH depletion in opposite directions affect cellular responses to EGF, with GLRXs dampening signaling and GSH depletion enhancing it. Next, we wished to study the effects of GLRX depletion, as well as compare the potency of GLRX-modulated EGF signaling with that controlled by TXN1.

### EGF-induced protein tyrosine phosphorylation in A431 cells is increased by knockdown of either GLRX1, GLRX2, or TXN1

To elucidate the effects of GLRXs or TXN1 knockdown on EGF-dependent phosphorylation, we used an EGFR inhibitor to more specifically analyze PTP-dependent dephosphorylation. Hence, EGF ligand was added for 4 min before addition of an EGFR inhibitor because, at the 4-min time point, the reductive reactivation process of PTP1B should have started. Any alteration of Tyr phosphorylation pattern under these conditions should thereby be mainly due to alterations in overall PTP activity. We also validated using immunohistochemistry that EGF treatment increased total Tyr phosphorylation levels and that any further phosphorylation was blocked upon addition of the EGFR inhibitor (fig. S1). When using this experimental setup, knocking down GLRX1 had no apparent effect on EGFR phosphorylation (fig. S2, A to C), which may be due to a compensatory increase in GSH levels (fig. S2D). To further confirm the role of GSH, we knocked down GLRX1 and simultaneously treated the cells with BSO to deplete GSH. As shown in fig. S2 (F and G), in the absence of GSH, EGFR phosphorylation increased irrespective of GLRX1 knockdown. This supports the idea that the lack of effect on EGFR phosphorylation under GLRX1 knockdown may be due to compensatory GSH synthesis. This finding, combined with the observation that TXN1, together with GSH, can support PTP1B activity (fig. S2E), further highlights the importance of GSH in regulating EGFR phosphorylation. We also confirmed biochemically that PTP1B undergoes glutathionylation under oxidative conditions induced by H_2_O_2_ and bicarbonate and demonstrated that glutathionylated PTP1B serves as a substrate for both GLRX1 and GLRX2 (fig. S2, H and I).

**Fig. 2.**
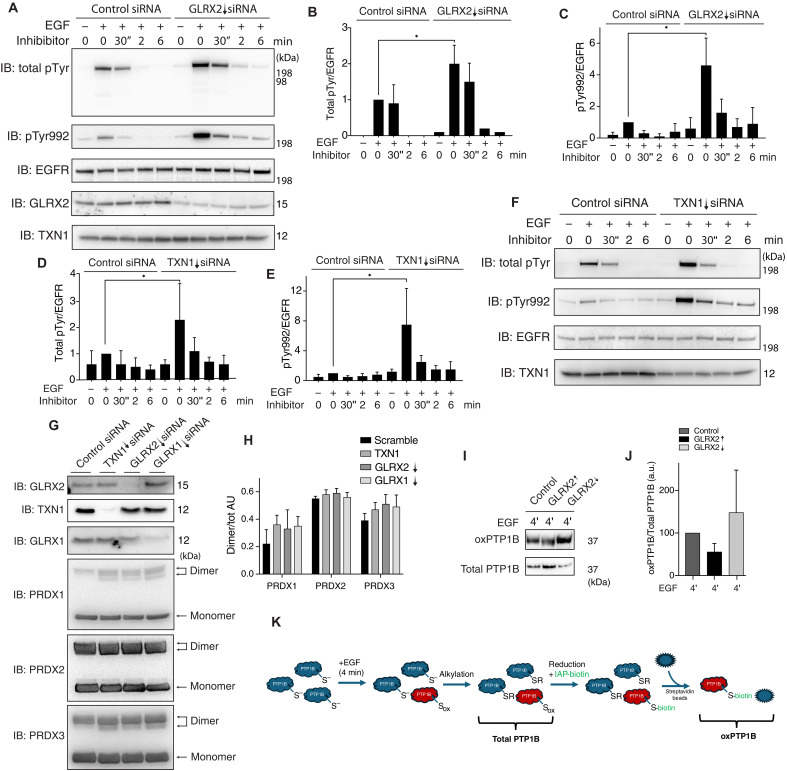
Knockdown of GLRX2 or TXN1 leads to an increase in tyrosine phosphorylation in A431 cells stimulated with EGF. A431 cells with knockdown of (**A**) GLRX2 or (**F**) TXN1, indicated with downward arrows, were serum starved before stimulation with EGF (100 ng/ml) for 4 min, after which EGFR receptor inhibitor (10 μM) was added. Cells were harvested at indicated time points, and receptor phosphorylation (total pTyr and pY992) was analyzed by immunoblotting. (**B**, **C**, **D**, and **E**) The densitometric signals were normalized for the intensity of the total expression of the EGFR. The same samples were used to confirm knockdown of TXN1 and GLRX2. Quantifications and densitometry of total pTyr and pTyr992 in respective experiments as shown in (A) and (D). The signals were normalized for the intensity of the total expression of the EGFR at 4 min. (**G** and **H**) Analyses of PRDX1, PRDX2 or PRDX3 dimer form versus total ratio for each PRDX isoform, in arbitrary units (Dimer/tot AU) after GLRX1, GLRX2, or TXN1 knockdown, as indicated. Bar graphs are determinations from immunoblots of three separate cell experiments [*n* = 3; means ± SE (error bars); *P < 0.05]. (**I**, **J**, and **K**) A431 cells with or without knockdown of GLRX2 or overexpression of GLRX2 were serum starved before stimulation with EGF (100 ng/ml). At the indicated times after EGF stimulation, cells were subjected to the modified cysteinyl-labeling assay using biotinylated iodoacetyl-PEG2-biotin for analysis of reversible PTP1B oxidation. Biotinylated proteins were purified using streptavidin-Sepharose beads and resolved by SDS-PAGE as illustrated in the scheme (K). Visualization was performed using antibodies against PTP1B, and control levels were determined from total cell lysate by SDS-PAGE and blotting against PTP1B. The densitometric signals were normalized for the intensity of PTP1B streptavidin-biotin signals over total amount of PTP1B, thus indicating the ratio of oxidized PTPB1B over total PTP1B (oxPTP1B/Total PTP1B), in arbitrary units (a.u.). Graphs are determinations from immunoblots of three (GLRX2) separate experiments [*n* = 4; means ± SE (error bars); *P < 0.05]. Data were analyzed using one-way analysis of variance followed by Bonferroni post hoc tests for multiple comparisons with GraphPad Prism.

Intriguingly, in cells where either GLRX2 (Fig. 2, A to C) or TXN1 (Fig. 2, D to F) had been knocked down, the EGFR dephosphorylation was significantly hampered as seen with the sustained extent of both total (Fig. 2, A, B, and D to F) and PTP1B site–specific (Fig. 2, A, C, E, and F) Tyr phosphorylation. Using a cysteinyl-labeling assay for detection of oxidized PTP1B in a cellular context, we also found that GLRX2 short hairpin RNA or overexpression can modulate the oxidation status of PTP1B in A431 cells (Fig. 2, I to K). The effects of GLRX1 (fig. S2, J and K) were less prominent, again probably due to the compensatory regulation of GSH (fig. S2D). To ensure that the effects of knockdown of TXN1 or GLRXs on PTP1B oxidation state and Tyr phosphorylation extent were not due to an increased overall oxidative pressure, we also analyzed the oxidation status of PRDX1, PRDX2, and PRDX3 in these cells. The most common method to visualize H_2_O_2_-induced PRDX oxidation is through nonreducing gel electrophoresis followed by Western blotting for PRDX monomers and dimers; the ratio of reduced monomers to oxidized dimers reflect the overall redox state of cells (*38*). Here, no differences in PRDX dimerization were observed upon knockdown of the redoxin proteins as compared to small interfering RNA (siRNA) control cells (Fig. 2, G and H), indicating that the global redox states of the cells were not perturbed, suggesting that both GLRXs and TXN1 more directly control PTP1B activity in cells. Thus, we next investigated potential direct intracellular interactions between PTP1B, TXN1, and GLRXs in conjunction with growth-factor signaling, using proximity ligation assay (PLA) for visualization of protein-protein interactions (*39*, *40*).

### PTP1B and GLRX1 are in proximity of each other within minutes after PDGF or H_2_O_2_ stimulation of hPTP1B-expressing MEFs

To first validate the specificity of the PLA method with our chosen anti-PTP1B antibodies, we used PTP1B-deficient (PTP1B^−/−^) mouse embryonic fibroblasts (MEFs) reconstituted with human PTP1B (hPTP1B) (*41*). The cells expressing hPTP1B were stained nicely for PTP1B, with a perinuclear localization as expected (*42*), as opposed to the parental PTP1B^−/−^ cells where no signal was detected (fig. S3A).

**Fig. 3.**
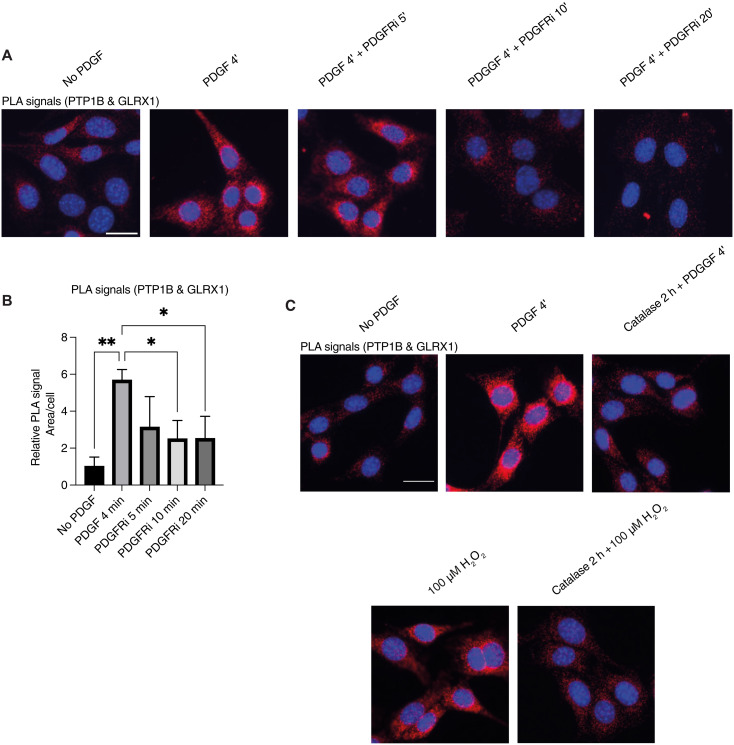
GLRX1 is in transient proximity to hPTP1B after stimulation with PDGF and their PDGF-triggered association is dependent upon H_2_O_2_. (**A**) MEF cells reconstituted with hPTP1B were grown on coverslips, serum starved, and stimulated with PDGF (50 ng/ml) for 4 min, and subsequently treated with 10 μM PDGFR inhibitor (PDGFRi) for 5, 10, and 20 min. Cells were fixed, permeabilized at the indicated times, and subsequently evaluated with primary antibodies from different species for hPTP1B (mouse) and GLRX1 (goat) to reveal protein-protein interactions in situ using PLA (red spots). Nuclei were labelled with 4′,6-diamidino-2-phenylindole (DAPI; blue). (**B**) Visualization and quantification were performed using fluorescent microscopy and ImageJ. Statistical differences in signal quantification are indicated in the bar graph (*P < 0.05; **P < 0.01); *n* ≥ 3. Error bars denote SEM. (**C**) MEF cells reconstituted with hPTP1B were grown on coverslips, serum starved, and stimulated with PDGF (50 ng/ml) for 4 min or with 100 μM H_2_O_2_ for 10 min, with and without catalase pretreatment (2 hours; 50 U/ml) as indicated. The images display results from one representative experiment, out of three independent experiments. Scale bars, 30 μm. Data were analyzed using one-way analysis of variance followed by Bonferroni post hoc tests for multiple comparisons with GraphPad Prism.

GLRX1 was detected in abundance in the MEF cells with an even cellular distribution; in contrast, GLRX1 was close to no signal in cells treated with siRNA (fig. S3B). We found that PLA signals using these antibodies, thus indicating direct GLRX1 and PTP1B interactions, could be detected upon addition of PDGF but only in the hPTP1B reconstituted cells (fig. S3C). With this validated model system, we subsequently used serum starved cells stimulated with PDGF (50 ng/ml) for 4 min before addition of a PDGFR inhibitor to characterize possible temporal dynamics of hPTP1B-GLRX1 interactions. As above, an EGFR inhibitor was used to more specifically characterize the EGFR signal turn-off dynamics and thereby focus on PTP1B-dependent effects (*43*). After 4 min of PDGF stimulation, there was a clear increase in fluorescent signal in the PLA compared to nonstimulated cells, demonstrating a PDGF-triggered interaction between hPTP1B and GLRX1, which decreased after 5 additional minutes of incubation with PDGFR inhibitor to be nearly gone after 10 min (Fig. 3, A and B).

It was previously shown that treatment of cells with H_2_O_2_ can inactivate PTP1B (*15*) as well as induce ligand-independent growth-factor phosphorylation (*44*). To evaluate whether the GLRX1-PTP1B interaction could also be H_2_O_2_ dependent, as it should be if linked to NOX activation and PTP1B regulation, starved cells were here pretreated with catalase for 2 hours before stimulation with PDGF. Notably, this diminished the PLA signal, showing that the transient GLRX1-PTP1B interaction was H_2_O_2_ dependent (Fig. 3C). Treating cells directly with 100 μM H_2_O_2_, without addition of growth factor, could also trigger an interaction between hPTP1B and GLRX1 that could be prevented by catalase pretreatment (2 hours) (Fig. 3C). These results revealed that PDGF stimulation triggered a H_2_O_2_-dependent transient protein-protein interaction between PTP1B and GLRX1, as detected with a highly specific PLA assay. Because this somewhat artificial model system used mouse cells either lacking or overexpressing hPTP1B, we next wished to analyze whether similar dynamics could be detected in human cells expressing their native levels of PTP1B and whether EGFR stimulation could give similar results.

### PTP1B is in proximity with GLRX1, GLRX2, and TXN1 within minutes after EGF stimulation of A431 cells

A431 cells were subjected to overnight starvation, followed by EGF stimulation and subsequent treatment with an EGFR inhibitor before PLA analyses. Antibody specificity was again validated, as shown in fig. S4.

**Fig. 4.**
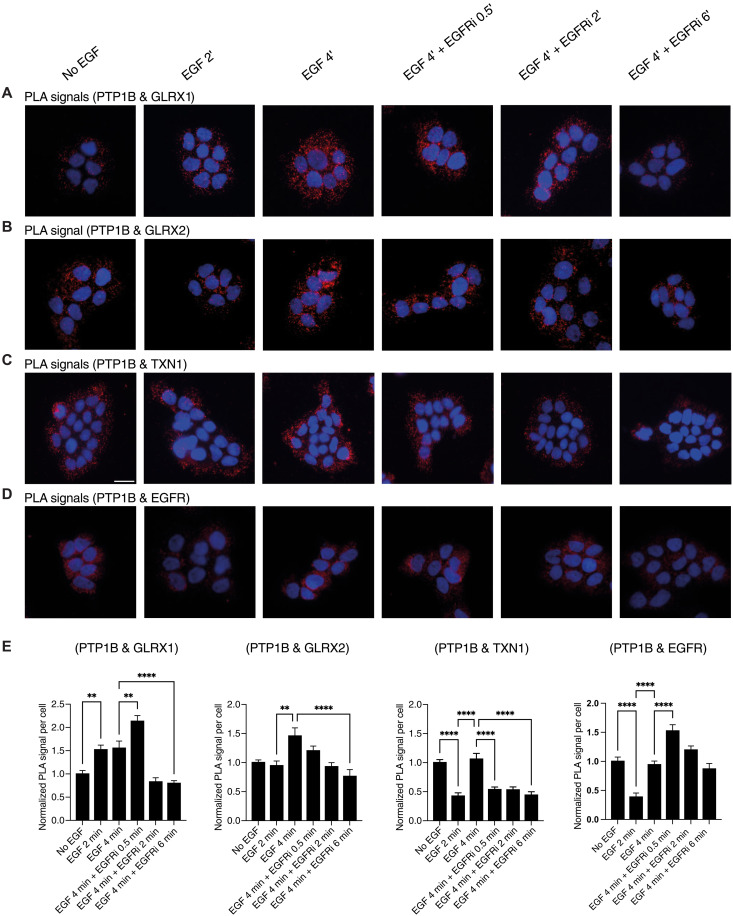
Both GLRX1 and TXN1 are in transient proximity with PTP1B after stimulation with EGF. A431 cells were starved for overnight, treated with EGF (100 ng/ml) for 2 and 4 min and subsequently with 10 μM EGFR inhibitor for 30 s, 2 min, and 6 min, as indicated. The cells were fixed and processed for PLA to determine protein interactions. Cells were stained with primary antibodies from different species against PTP1B (mouse) together with antibodies against (**A**) GLRX1 (goat), (**B**) GLRX2 (rabbit), (**C**) TXN1 (rabbit), or (**D**) EGFR (goat) to reveal protein-protein interaction in situ using the technique of PLA. The cells were fixed and processed for PLA to determine the direct interactions. Red spots indicate protein interactions with nuclei counterstained with DAPI (blue). (**E**) Bar graphs with quantifications and statistics of PLA signals (**P < 0.01; ****P < 0.0005); *n* ≥ 3 (more than 500 cells per experiment). Error bars denote SEM. Scale bar, 30 μm. Data were analyzed using one-way analysis of variance followed by Bonferroni post hoc tests for multiple comparisons with GraphPad Prism.

A strong transiently induced PLA signal for GLRX1 being in proximity with PTP1B was observed up to 4 min of EGF stimulation, which subsequently diminished after EGFR inhibitor treatment (Fig. 4A). Similarly to GLRX1, a PLA signal for either GLRX2 or TXN1 interacting with PTP1B, respectively, was transiently enhanced by EGF stimulation (Fig. 4, B and C). In sharp contrast, PLA signals for the proximity of EGFR with PTP1B transiently decreased upon EGF treatment, with a lower signal seen 2 min after EGFR stimulation, subsequently rebounding to initial levels after 4 min (Fig. 4D). This suggests a critical time window after EGFR stimulation when PTP1B (presumably then in an oxidized state) interacts with both the GLRX- and TXN1-based reducing systems. This temporal information hence provides detailed insights into the dynamic interplays between EGFR signaling and the redox regulation of its signaling through PTP1B, GLRXs, and TXN1.

### Pure PTP1B inactivation in vitro by H_2_O_2_ and bicarbonate is abrogated by GSH through clearance of H_2_O_2_

**Fig. 5.**
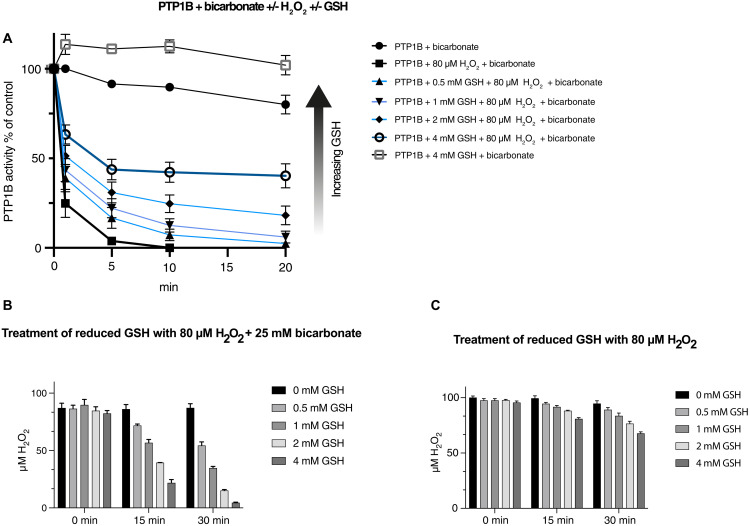
GSH protects PTP1B from H_2_O_2_/bicarbonate-dependent inactivation by clearance of H_2_O_2_. (**A**) Recombinant PTP1B (600 nM) was treated with H_2_O_2_ /bicarbonate (80 μM/25 mM) in the presence of increasing concentration of GSH (0.5 to 4 mM, blue lines). All incubations were performed in 50 mM Hepes, 100 mM NaCl buffer (pH 7.4), 0.1% bovine serum albumin (BSA), and 2 mM sodium azide, with subsequent measurements of PTP activity at the indicated times. PTP1B activities are presented as percentages of untreated control. Data points represent means ± SEM (*n* = 3). (**B**) GSH (0 to 4 mM) was incubated with 80 μM H_2_O_2_ and 25 mM bicarbonate or (**C**) 80 μM H_2_O_2_ alone. The concentration of H_2_O_2_ in the different samples in (B) and (C) was determined at 15 and 30 min by ferrous oxidation of xylenol orange assay, performed as described previously (*1*). Error bars represent means ± SEM; *n* = 3. Data were analyzed using one-way analysis of variance followed by Bonferroni post hoc tests for multiple comparisons with GraphPad Prism.

To further characterize the possible underlying molecular mechanisms for the cellular findings showing regulation of PTP1B by both GLRXs and TXN1, we next characterized in more detail their biochemical PTP1B activity support using recombinant proteins. Inactivation of the PTP1B catalytic domain by H_2_O_2_ is potentiated by addition of bicarbonate through the formation of peroxymonocarbonate (*13*), but the effects of GLRXs and/or GSH on PTP1B activity during H_2_O_2_/bicarbonate treatment in that system are not yet known. GSH reacts slowly with H_2_O_2_, and, therefore, we first assessed the effects of GSH addition on PTP1B activity during treatment with H_2_O_2_ in the presence of bicarbonate, using 80 μM H_2_O_2_ combined with 25 mM bicarbonate. As we (*14*) and others (*13*) have shown previously, H_2_O_2_ in combination with bicarbonate potently inactivates PTP1B. We found here that the presence of GSH in this inactivation assay dose-dependently slowed down the PTP1B inactivation (Fig. 5A), which coincided with the clearance of H_2_O_2_ (Fig. 5B).

Omission of bicarbonate showed a marked decrease in GSH-mediated H_2_O_2_ clearance, suggesting that bicarbonate facilitates the general reactivity of H_2_O_2_ with thiols (Fig. 5C). Considering this finding, we next determined the protection of PTP1B by TXN1 and GLRXs, alone or in combination and with or without the addition of GSH.

### GLRXs in combination with TXN1 and GSH potently protect PTP1B from H_2_O_2_/bicarbonate-mediated inactivation and can reactivate oxidized PTP1B

**Fig. 6.**
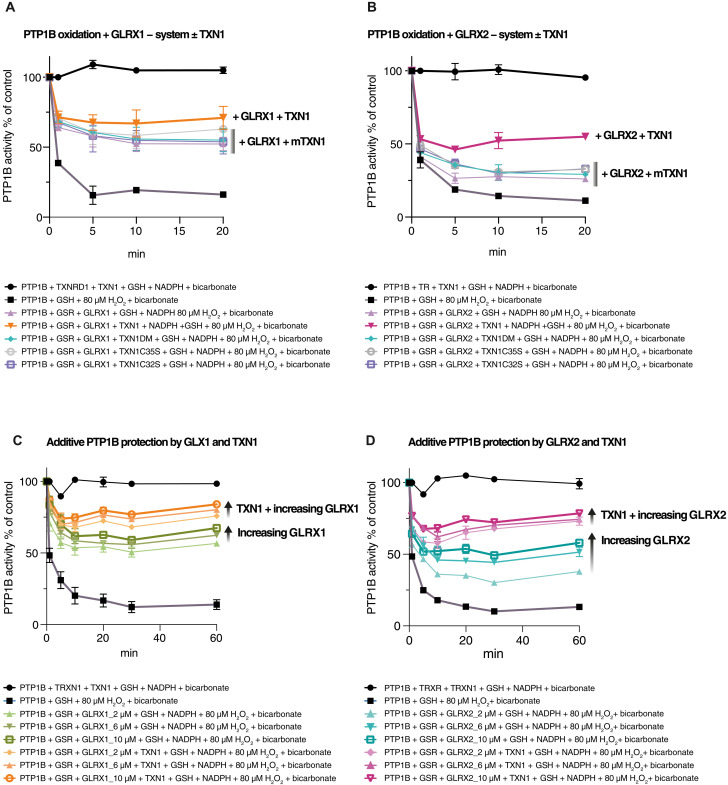
Cross-talk between GLRX1, GLRX2, and TXNRD1 in protection of PTP1B activity from H_2_O_2_/bicarbonate-mediated inactivation in the presence of GSH. (**A** and **B**) Active PTP1B was treated with H_2_O_2_ (80 μM) and bicarbonate (25 mM) in the presence of 2 mM GSH and enzyme components as indicated [10 μM GLRX1 in (A) or 10 μM GLRX 2 in (B) and NADPH, TXNRD1, or GSR as indicated], together with either wild type TXN1 [yellow line in (A) and pink line in (B)] or enzymatically inactive mutant TXN1 variants [C32S, C35S, or double mutant (DM) at 10 μM] collectively indicated as mutant TXN (mTXN1) in the graph. (**C** and **D**) PTP1B was treated with H_2_O_2_ (80 μM) and bicarbonate (25 mM) in the presence of 2 mM GSH and GLRXs (2 to 10 μM), with GLRX1 (green lines) in (C) or GLRX2 (blue lines) in (D), together with NADPH, TXNRD1/GSR, and/or wild type TXN1 (10 μM) as indicated [yellow lines in (C) or pink lines in (D)]. Measurements of PTP activity were performed at the indicated times points (*n* = 3 ± SEM). At the 20 min time point, the activities indicated with differently colored symbols are statistically different from each other in (B) to (D) (*P* < 0.05) but did not reach statistically significance difference in (A). Data were analyzed using one-way analysis of variance followed by Bonferroni post hoc tests for multiple comparisons with GraphPad Prism.

Recently, we used pure protein preparations to characterize the effects of the thioredoxin system on PTP1B activity upon H_2_O_2_ treatment with (*14*) and without (*34*) bicarbonate. In the thioredoxin system, TXNRD1 is considered the main enzyme reducing TXN1 both in vitro and in vivo, but a prior study suggested that GSH and GLRXs can act as backup systems for reduction of TXN1 (*45*). Considering our findings above showing that both GLRXs and TXN1 can support cellular PTP1B activity, we here further explored this concept in vitro using pure protein preparations. In the presence of GSH, either GLRX1 (Fig. 6A) or GLRX2 (Fig. 6B) protected PTP1B from H_2_O_2_/bicarbonate-dependent inactivation, and, intriguingly, the addition of TXN1 potentiated this protection also in the absence of TXNRD1 (Fig. 6, A and B).

Neither of the enzymatically inactive mutant variants of TXN1 (C32S, C35S, or the active site double mutant) further protected PTP1B from oxidation, above the protection seen with solely the GLRX1 (Fig. 6A) or GLRX2 (Fig. 6B) systems. Further experiments revealed an additive protection of PTP1B, whereby increasing concentrations of GLRXs increased the protection of PTP1B, above the protection seen by addition of TXN1, which was a phenomenon seen with both GLRX1 (Fig. 6C) and GLRX2 (Fig. 6D).

**Fig. 7.**
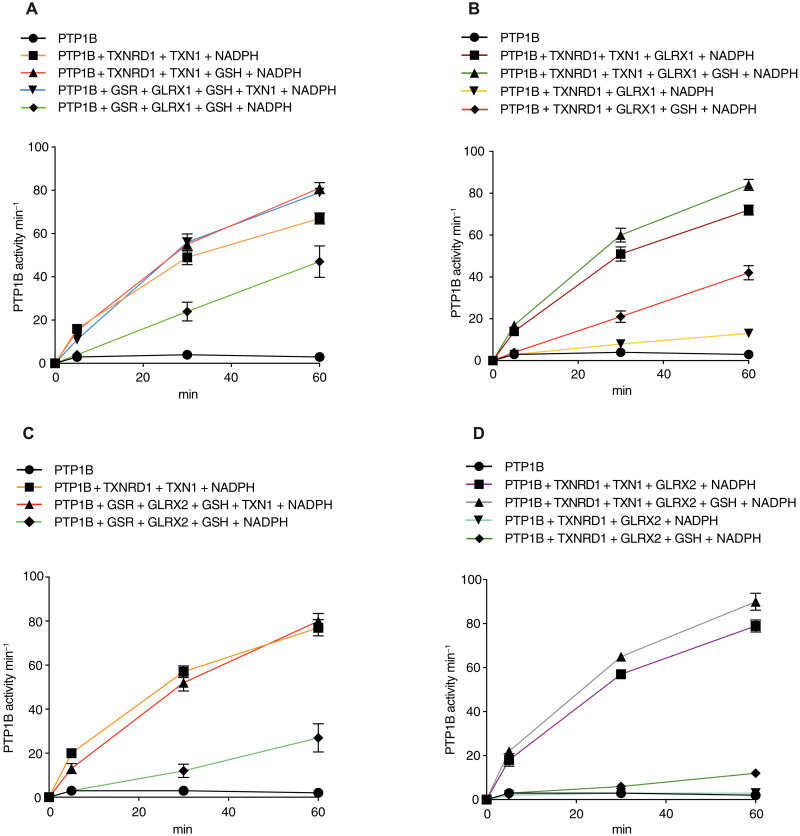
Reactivation of preoxidized PTP1B by the GSH/GLRX1/TXN1 systems. (**A** to **D**) Reduced PTP1B was treated with 300 μM H_2_O_2_ and 4 mM GSH for 30 min and then with catalase, followed by reactivation assessments with different combinations of TXNRD1 (250 nM), NADPH (300 μM), TXN1 (10 μM), GSR (30 nM), GSH 2 mM, GLRX1 (2 μM), or GLRX 2 (2 μM), as indicated. After 5, 30, and 60 min of incubations at 22°C, samples were analyzed for PTP activity (*n* = 3 ± SEM). At the final time point, the activities indicated with differently colored symbols are statistically different compared to control. Data were analyzed using one-way analysis of variance followed by Bonferroni post hoc tests for multiple comparisons with GraphPad Prism.

In previous work, we (*46*) and others (*25*) have shown that the thioredoxin system not only can preserve PTP1B activity upon addition of H_2_O_2_/bicarbonate but also can reactivate PTP1B that has first been preoxidized. Therefore, we next tested whether the combined GSH/TXN-system could also cooperate in reactivation of preoxidized PTP1B. After oxidation of PTP1B with H_2_O_2_ in presence of GSH and subsequent clearance of H_2_O_2_ using catalase, inactivated PTP1B was exposed to either the thioredoxin system alone, GLRXs and GSH alone, or the combined GLRX/TXN/GSH system. The GLRXs and GSH together with TXN1, but in the absence of TXNR1, were able to reactivate PTP1B to the same extent as the complete thioredoxin system, irrespectively if GLRX1 (Fig. 7A) or GLRX2 (Fig. 7C) was used. However, combination of either GLRX1 or GLRX2 together with TXNRD1, in the absence of TXN1, was unable to recover PTP1B activity. In contrast, when reduced GSH was included together with either the thioredoxin system or the GLRXs, PTP1B activity was well recovered (Fig. 7, B and C). In comparison to the complete thioredoxin system or the GLRX1 system with addition of TXN1, the GLRXs alone were the least potent in reactivating PTP1B (diamond symbols and green lines in Fig. 7, A and B). GLRX1 or GLRX2 were not as efficient as the thioredoxin system in reduction of insulin disulfides, and no cross-talk was found between GLRXs/GSH and TXN1 in that well-defined disulfide reduction assay (fig. S5). It is thus likely that, when GLRXs/GSH and TXN1 act in tandem for the protection and activation of PTP1B, this can be explained by the fact that oxidized species of PTP1B involve more motifs than a mere disulfide, including a sulfenylamide and glutathionylated species, where GLRXs and TXN1 should have different potencies in their reductive activities.

## DISCUSSION

In this study, we characterized the temporal regulation of RTK signaling in cells through the redox control of PTP1B activity. We identified a previously unrecognized cross-talk between the GLRXs/GSH and TXN1 in regulation of PTP1B activity, which was further characterized in vitro using pure enzyme systems. The results demonstrate that RTK signaling, as shown with stimulation of PDGFR and EGFR using PDGF and EGF, respectively, is intimately linked with the cellular redox pathways. We also found that GLRXs and TXN1 should be considered as critical enzymes for control of Tyr phosphorylation cascades through modulation of cellular PTP1B activity. Several implications of these findings will here be further discussed.

### Balance between oxidation and reduction of PTP1B in control of RTK signaling

The interplay between H_2_O_2_ and CO_2_/bicarbonate was recently recognized as important for control of redox regulated enzymes and should be considered with regard to oxidative pathways controlling enzyme activities (*47*). This includes PTP1B regulation through redox control. The overall balance between oxidative and reductive pathways must be considered for a full understanding of PTP1B activities in relation to RTK signaling. Onset of growth-factor signaling through RTKs has long been known to rely on the controlled production of membrane-localized H_2_O_2_ to temporarily turn off PTP activity through reversible oxidation (*15*, *44*, *48*). This implies that the reduction of oxidized PTPs, with regain of PTP activity and thus termination of the corresponding RTK signal, is important. In other words, the overall balance between both oxidative and reductive pathways regulating RTK signaling will ultimately determine the overall Tyr phosphorylation state. The reductive pathways have, however, been far less studied in this context, although it is well-known that PTPs such as PTP1B are subject to reduction by both the TXN1 and GLRXs (see Introduction). Here, we have characterized the time-dependent cross-talk between the GLRXs/GSH and TXN1 systems in control of Tyr phosphorylation cascades as triggered by PDGF or EGF. We were initially surprised to find such notable effects by overexpression or knockdown of the GLRXs or by BSO treatment, hence revealing a strong regulatory role of GSH, GLRX1, and GLRX2, in addition to TXN1, in PTP1B regulation. The exception was the notable lack of effect upon GLRX1 knockdown, which we suggest is explained by the resulting increase in cellular GSH levels, which should be due to the recently discovered GLRX1-mediated control of *S*-glutathionylation of OTUB1 and system xC^−^, leading to increased GSH levels in the absence of GLRX1 (*49*). Together, our results illustrate how important GSH, GLRX1, GLRX2, and TXN1 are in redox control of PTP1B and most likely also other PTPs and thus in control of RTK signaling. Because dysfunctional RTK signaling plays a substantial role in many diseases, including cancer, the involvement of PTPs in these pathologies suggests them to be attractive targets for drug development (*1*, *50*). We conclude that any interventions of PTP activities for therapeutic purposes should also consider the redox regulation of these systems. Based on our findings herein, we can draw some general conclusions regarding the intracellular redox control of PTP1B activity when linked to RTK signaling as triggered by EGF or PDGF.

### Intracellular dynamics of PTP1B redox control

**Fig. 8.**
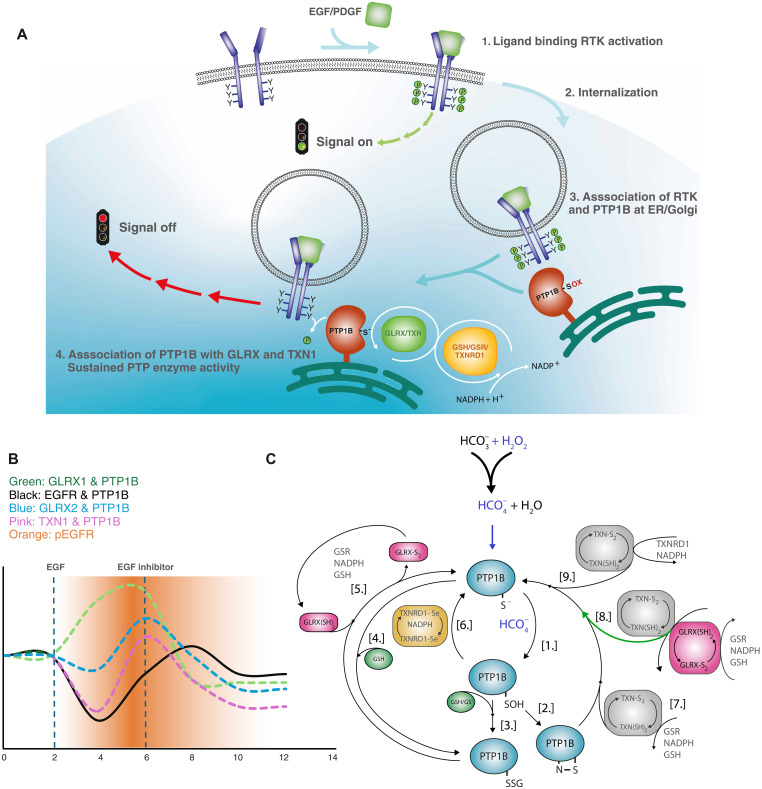
Proposed redox regulation of RTK and PTP1B activities by the TXN/GLRX system. (**A**) Ligand binding to the RTK receptor induces transphosphorylation (step 1, signal on) and oxidation of PTP1B through NOX activation (not shown in the scheme). Ligand-bound EGFR complexes are then internalized (step 2) and at the perinuclear area associates with oxidized PTP1B that becomes reactivated by the GLRX /TXN systems acting in tandem (step 3), resulting in dephosphorylation of the RTK terminating the signal (step 4, signal off). (**B**) Graphical time scheme, where the lines indicate the degree of interaction between PTP1B, GLRX1, TXN1, and EGFR over time (in minutes). The degree of EGFR phosphorylation is shown as a color gradient. (**C**) Model scheme for the different reductive steps by which the TXN and GLRX systems can regulate the activity of PTP1B. The bold green arrow shows the reaction of GLRX and TXN1 in combination with GSH reducing the sulfenylamide form of PTP1B. Further details of the schemes are discussed in the text.

We had earlier found that stimulation with EGF ligand induces oxidation of PTP1B with a peak at 2 min followed by the onset of phosphorylation (*14*). This guided us herein for the planning of our studies on the timing control of the Tyr phosphorylation cascades upon redox modulation. In Fig. 8, we attempt to graphically summarize our main findings and proposed series of events, specifically with regard to regulation of RTK signaling upon stimulation of cells with EGF or PDGF through redox control of PTP1B. First, the growth-factor ligand binds to its respective RTK at the cell surface (Fig. 8A, step 1), which triggers production of H_2_O_2_ that, in the presence of bicarbonate, will lead to PTP1B oxidation (as discussed above; not depicted in Fig. 8A), thus triggering the downstream Tyr phosphorylation cascades (Fig. 8A, “signal on”). The RTK is then rapidly, within 1 to 2 min, internalized (Fig. 8A, step 2) and translocated to the perinuclear area, where it associates with PTP1B (Fig. 8A, step 3) that is being reduced and activated by the reductive systems involving both GLRXs and TXN1 (Fig. 8A, step 4). Upon reduction and reactivation of PTP1B by these enzyme systems, active PTP1B directly interacts with the RTK to exert its dephosphorylating activity, leading to dephosphorylation of the receptor and termination of the signaling cascade (Fig. 8A, “signal off”).

Based on the dynamics in the Tyr phosphorylation patterns observed in immunoblots and PLA signals (while acknowledging the limitations of these techniques), we suggest that the temporally resolved protein-protein interactions between PTP1B and GLRX1 (Fig. 8B, green line), between PTP1B and GLRX2 (Fig. 8B, blue line), and between PTP1B and TXN1 (Fig. 8B, pink line) appear to occur within a time frame consistent with the reduction and activation of PTP1B by these redoxins, followed by the subsequent dephosphorylation of EGFR. The PTP1B and TXN1 interactions as detected herein are in agreement with previous studies where TXN1 was found to be an important regulator of PTP1B and of growth-factor signaling (*15*, *25*, *34*, *46*, *51*), while the control of PTP1B by GLRX1 and GLRX2 has previously not been extensively studied in a cellular context. Notably, we also found that the interaction of PTP1B with EGFR first disappeared upon EGF addition and then increased again, which should reflect the PTP1B-catalyzed dephosphorylation of the receptor (Fig. 8B, black line). Interactions between PTP1B and EGFR were previously suggested to occur either when EGFR is internalized (*52*), through contact sites between the ER membrane and the plasma membrane (*53*), or through relocalization of proteolytically cleaved PTP1B (*42*). Here, we found the PTP1B/EGFR-derived PLA signals to be localized mainly to the perinuclear area, indicative of the ER membrane, although all three previously described interaction modes could be compatible with our results. The interaction between PTP1B and EGFR as detected here is in agreement with a previous study where the PLA technique was combined with a substrate-trapping mutagenesis approach to identify unknown substrates of PTP1B in HER2-positive and Herceptin-resistant cells. In that study, the authors identified interactions with known substrates, such as EGFR, as well as several previously unknown targets. However, none of the redoxins were found among the targets, probably as a result of the biotinylation approach in combination with the slow turnover of the mutated PTP1B constructs, with the methodology specifically aimed at identifying phosphorylated protein substrates for PTP1B (*54*).

The effectiveness of PTP1B in RTK signal termination was highlighted in a previous study where the authors used a photoswitchable variant of PTP1B; the most profound effects on dephosphorylation of the insulin receptor were seen when a nuclear localized protein, as opposed to a variant targeted to the plasma membrane, was transiently irradiated and thus activated (*55*). The same study concluded that only modest changes in PTP1B activity had rather large effects on overall Tyr phosphorylation status. Those findings agree with the marked effects on Tyr phosphorylation levels as seen by us when modulating the GLRX1, GLRX2, or TXN1 expression levels or the GSH levels. Similarly, strong effects when TXN1 was either overexpressed or knocked down were found by the Tonks group with regard to insulin signaling and the insulin receptor phosphorylation status (*51*), but the large effects of GLRX1 or GLRX2 modulation as seen here have, to our knowledge, not been reported previously.

A few studies have previously characterized glutathionylation of PTPs in cellular systems. An early study showed that PTP1B was glutathionylated in macrophages upon an adenosine 5′-diphosphate–stimulated oxidative burst (*20*), and, in another study, modulation of the GLRX system during PDGF stimulation affected the activity of LMWPTP and downstream phosphorylation events (*35*, *56*). That study suggests that also other reversibly oxidized PTPs are under control of the two antioxidant systems studied herein, which thus ultimately modulate the corresponding phosphorylation responses. Differential sensitivity toward the different reducing systems of PTPs has been shown for TRP14 (TXNDC17) and TXN1, both found to reduce PTP1B, but not SHP2 (Src-homology 2 domain-containing phosphatase 2; encoded by *PTPN11*) (*46*). Most likely, also, different GLRXs could show different efficacy in reduction of specific PTPs. Intriguingly, GLRX1 and GLRX2 are known to have different affinities toward glutathionylated proteins (*57*). Furthermore, GLRX2 is not solely dependent on reduced GSH but can also be reduced by thioredoxin reductase (*58*), which may help to explain the differences in phosphorylation pattern seen in BSO-treated cells between GLRX1 and GLRX2 overexpression, with the latter not being as affected by BSO treatment. A recent study also found glutathionylation of PTP1B after EGF stimulation in lung cancer H292 cells; the sulfenylamide form was suggested to protect cysteine residues from overoxidation and supposed to readily react with GSH to form glutathionylated cysteines (*18*, *59*). Herein, we wished to exclude that the knockdown of GLRX1, GLRX2, or TXN1 would have affected the overall cellular H_2_O_2_ clearance, so that the PTP1B effects seen in cells would solely have been indirect due to an overall increased oxidative pressure, whereby we analyzed the oxidation states of the PRDX1, PRDX2, and PRDX3. These are the major H_2_O_2_ scavenging peroxiredoxins in cells, and, upon increased oxidative stress, they typically present increased levels of their dimeric forms. However, we noted no difference in dimer over total ratio of either PRDX isoenzyme, suggesting that the change in Tyr phosphorylation cascades upon modulation of GLRXs or TXN1 should mostly likely have been due to modulations of their direct capacities in reduction and reactivation of PTP1B.

The interactions between PTP1B and GLRX1 as found in our PLA analyses occurred earlier than the interactions of PTP1B with either GLRX2 or TXN1. This differential temporal pattern might be reflective of the fact that PTP1B can be oxidized into a mixture of forms, which are then uniquely targeted for reduction by either GLRX1, GLRX2, or TXN1. The findings thus suggest that the forms of oxidized PTP1B that can be reduced by GLRX1 are the ones to be reduced first after PTP1B oxidation subsequent to RTK stimulation. The different aspects of PTP1B activity support by GSH, GLRX1, GLRX2, and/or TXN1 were here also studied at further depth using pure enzymes, as briefly discussed next.

### Different oxidized PTP1B species and their reduction by GLRXs, GSH, and TXN1

Based on our results with pure proteins, we suggest that GLRX1, GLRX2, and TXN1, with an impact of GSH, support the activity of PTP1B through different specific reactions, as graphically illustrated by the model scheme in Fig. 8C. First, we find that inclusion of bicarbonate together with H_2_O_2_ can rapidly oxidize PTP1B, which likely occurs through formation of peroxymonocarbonate (HCO_4_^−^) that first oxidizes the active site Cys residue of PTP1B to a sulfenic acid (Fig. 8C, step 1), which, subsequently, is either converted into the sulfenylamide form of PTP1B (Fig. 8C, step 2) or becomes glutathionylated upon reaction with GSH (Fig. 8C, step 3). Based on our prior studies (see Introduction), we believe that both the sulfenic acid and sulfenylamide forms of PTP1B can be reduced directly by the TXN1 system (Fig. 8C, reactions 6 to 9), while the glutathionylated forms of PTP1B should preferably be reduced by GLRX1 or GLRX2 (Fig. 8C, reaction 5).

As also noted above, with the knockdown of GLRX1, we noted no difference in Tyr phosphorylation compared to control cells, in contrast to the GLRX1 overexpression where a notable effect was seen. Because knockdown of GLRX1 leads to higher GSH levels in cells, as found here and recently shown by the group of Janssen-Heininger to be through control of xC^−^ and cystine uptake (*49*), the higher GSH levels could have increased both the H_2_O_2_-scavenging capacity of GSH (either directly or through GPXs), as well as the reductive capacity of the GLRXs/TXN1-linked effects of GSH in reduction of PTP1B (Figs. 5 and 6), as here summarized graphically (Fig. 8C, reactions 5 and 8). As noted above, GSH can also react directly with the sulfenic acid form of PTP1B (Fig. 8C, reaction 3) or the sulfenylamide form, as has been suggested previously (*21*, *60*). Reactivation of oxidized PTP1B directly by GSH can occur, but very slowly, as shown by several laboratories (*15*, *25*, *51*, *60*). Also, the complete GLRX system, coupled with GSH, GSR, and NADPH (Fig. 8C, reaction 5) seems to be only moderately effective in comparison to the TXN1 system as coupled with TXNRD1 and NADPH (Fig. 8C, reaction 9), which was also noted previously (*15*, *21*). Here, we found a differential preference regarding the exact PTP1B substrate form and the efficiency between the two reductive GLRX1/GLRX2 versus TXN1 systems, for instance, in contrast to the high potency of the thioredoxin system in reduction of oxidized insulin where the GLRXs were comparatively rather inefficient. In reactivation or protection of PTP1B exposed to H_2_O_2_/bicarbonate, a strong cross-talk was revealed between TXN1 and GLRX1/GLRX2 coupled with GSH. That particular additive and protective effect of TXN1 likely involved TXN1 and GLRX1 targeting different oxidant forms of PTP1B, such as the sulfenic acid, sulfenylamide, or the glutathionylated forms, as depicted in our suggested model scheme (Fig. 8C, reactions 5, 7, and 8).

### Limitations of this study

Several prior studies have demonstrated oxidative modifications of PTP1B (*14*, *15*), including glutathionylation (*20*, *21*, *26*). Here, we thus set out to study the impact of cellular reducing systems on PTP1B function in cells. We found that knockdown or overexpression of GLRX1, GLRX2, or TXN1 affected RTK signaling and PTP1B-regulated phosphorylation cascades in a direction compatible with their efficacy in supporting PTP1B activities in pure enzyme systems. The PLA results neatly showed that the redoxins are in transient close proximity to PTP1B during growth-factor signaling. The timing of that interaction matches the presumed time window for reduction and reactivation of PTP1B upon RTK stimulation, in agreement with previous publications (*14*, *26*), although the PLA characterization does not directly prove a direct reduction of the active site of PTP1B. The EGF- and PDGF-induced increases in total receptor– and PTP1B site–specific receptor phosphorylation patterns with their respective modulation upon altered GLRX1, GLRX2, and TXN1 cellular levels are also only indirect measurements, but, again, in strong agreement with a model proposing a direct regulation by these redoxins of PTP1B activity in cells. In further support of that notion is the GLRX2 status-dependent modulation of reversible PTP1B oxidation in cells, as analyzed using the cysteinyl-labeling assay. Thus, even if we did not directly demonstrate involvement of PTP1B glutathionylation within the cellular context, because GLRXs efficiently deglutathionylate proteins, their impact on PTP1B oxidation most likely involve modulation of the extent of PTP1B glutathionylation. However, GLRXs and TXN1 may also act indirectly through other protein targets, including the possibility of directly reducing oxidized motifs in EGFR, because EGFR has earlier been shown to be amenable to both glutathionylation and persulfidation (*61*). It should, furthermore, be noted that regulation of RTK phosphorylation is multifaceted, involving several PTPs in addition to PTP1B. For example, TCPTP can also dephosphorylate EGFR (*62*) and PDGFR can be regulated by several additional PTPs, including SHP2 (*63*) and DEP-1 (*64*). In the context of PDGF and vascular endothelial growth factor signaling, LMW-PTP was shown to be glutathionylated and could hence also be regulated by GLRXs (*35*, *56*). Previous work by others using pure enzyme systems characterized glutathionylation patterns of PTP1B, with the kinetic evaluation of PTP1B and SHP2 preoxidized with H_2_O_2_ showing differential sensitivity to reduction by GSH (*25*). Rate constants for the initial reaction of oxidized PTP1B with GSH as followed by a slow reactivating step suggested that glutathionylated PTP1B could easily accumulate upon a burst of H_2_O_2_, as suggested earlier (*20*), and glutathionylated PTP1B is formed upon EGF stimulation of H292 cells (*26*). The exact states of PTP1B to be reductively activated during RTK signaling, either directly or indirectly, as well as the identity of possible additional PTPs regulated within the cellular context by GLRX1, GLRX2, and TXN1, should be the focus of forthcoming studies.

### Potential importance of PTP1B redox control for RTK signaling

Together, in this study, we have characterized the effects of the two main cytosolic antioxidant enzyme systems, GLRXs and TXN1, in regulation of PTP1B during EGFR and PDGFR signaling. The EGF- and PDGF-triggered Tyr phosphorylation cascades depend on the interplay between the respective RTKs and PTP1B, with PTP1B, in turn, being potently regulated through redox control exerted by the GLRXs and TXN1. For long, PTPs such as PTP1B were only regarded as housekeeping enzymes. However, today, it is clear that PTPs are specific regulators of signaling and function, acting in concert with RTKs. The potent cross-talk between GLRXs and TXN1 in redox regulation of PTP1B as found here shows that redox regulation of PTP1B through GLRXs and TXN1 should be recognized as potently regulating RTK signaling efficacy. Several diseases are related to either increased oxidative stress (*65*) or reductive stress with activation of NRF2 target genes and increased antioxidant pathways (*66*), as the drivers of disease (*67*). Based on the present study, we propose that alterations in activities of the main cytosolic reductive enzyme systems, involving GSH, GLRX1, GLRX2, and TXN1, should also be viewed in the context of their capacity of directly regulating the activities of PTP1B and likely also other PTPs, thereby ultimately regulating cellular responses and disease phenotypes related to RTK signaling.

## MATERIALS AND METHODS

### Recombinant proteins

The PTP1B catalytic domain (1-322) was used for the in vitro assays expressed under reducing conditions, as described before (*34*, *68*). A buffer exchange (Zeba Spin Desalting Columns from Thermo Fisher Scientific, catalog no. 87766) was used to remove any present reductants in the preparations. Subsequent to the buffer exchange, protein concentrations were determined using Bradford reagents (Bio-Rad). Expression of recombinant human TXN1, TXNRD1, GSR, GLRX1, and GLRX2 proteins was performed, as previously described (*69*).

### Inactivation of recombinant PTP1B using bicarbonate/H_2_O_2_

Reduced and desalted recombinant PTP1B (2.7 μM) was exposed to 300 μM H_2_O_2_ together with 25 mM sodium bicarbonate (pH 7.4) for 30 min in assay buffer [50 mM Hepes, 100 mM NaCl, and 0.1 mM EDTA buffer (pH 7.4) containing 0.05% bovine serum albumin (BSA) and 1 mM sodium azide] with and without 4 mM GSH. After the inactivation, H_2_O_2_ was cleared using catalase (20 U/ml). Reversibly oxidized PTP1B was then exposed to combinations of TXNRD1/TXN1 and NADPH (Sigma-Aldrich, N7505-100MG), and PTP1B activity was determined at 5, 15, and 60 min.

### PTP1B activity measurement

Determination of PTP1B activity was performed by using a chromogenic substrate 4-nitrophenyl phosphate (Sigma-Aldrich, P4744-1G) (15 mM), as described previously (*70*). Substrate turnover was calculated using absorbance curves and a 4-nitrophenol standard curve as determined using spectrophotometric analysis at 410 nm and 22°C [Infinite M200 Pro plate reader (Tecan)]. Reduced PTP1B (600 nM) was preincubated for 20 min in 50 mM Hepes and 100 mM NaCl buffer (pH 7.4) containing 0.1 mM EDTA, 0.1% BSA, and 1 mM sodium azide with the indicated concentrations of TXN1, TXNDR1, GSR, GLRX1, and GLRX2 NADPH (Sigma-Aldrich, N7505-100MG). Sodium azide was used to inhibit any trace amounts of catalase. For control, each treatment condition was compared to fully reduced PTP1B in the presence of the complete TXN1-coupled system and/or bicarbonate/H_2_O_2_.

### Determination of kinetic parameters of TXN1 activity

Kinetic measurement was used to measure the activity of recombinant TXN1. Experiments were performed in tris-EDTA buffer (pH 7.5) using pure control TXNRD1 (∼250 nM final), GSR (30 nM), GLRX1/2 (2 μM), GSH 4 mM, and TXN1 (0 to 10 μM). NADPH (300 μM) consumption was determined by analysis of decrease of absorbance of 340 nm at the initial linear phase.

### Determination of H_2_O_2_ concentration

GSH with and without PTP1B [in 50 mM Hepes, 100 mM NaCl, 0.05% BSA, and 2 mM sodium azide (pH 7.4)] was treated with 80 μM H_2_O_2_, and decrease in H_2_O_2_ was determined using the ferrous oxidation of xylenol orange assay (*71*).

### Cell culture

The epidermoid carcinoma cell line A431 (American Type Culture Collection) or MEFs were cultured in DMEM (5% CO_2_) + 10% (v/v) fetal bovine serum (FBS), 2 mM l-glutamine, penicillin, and streptomycin. No “additional selenium source” was added. EGF ligand (100 ng/ml; R&D Systems, no. 236-EG-200) stimulations, at indicated time points, were performed in overnight-starved (0.1% FBS) cells transiently transfected at 90% confluency. For overexpression, either empty vector control, GLRX1, or GLRX2 plasmids were transfected by mixing vector DNA (1.28 μg per 540,000 cells) in 500 μl of Opti-MEM with Lipofectamine 3000 reagent following manufacturing information. For down-regulation, scrambled negative control siRNA duplex, three different gene-specific 27-mer siRNA duplexes for GLRX1 and GLRX2 were transfected using Lipofectamine RNAiMAX in 500 μl of Opti-MEM following the manufacturer’s instruction. The transfection mixture was added to the cells, and, after 8 hours of starvation, medium was added overnight. For inhibition of EGFR phosphorylation, 10 μM EGFR inhibitor was used (Sigma-Aldrich, no. 324674-1MG).

### Analysis of protein tyrosine phosphorylation, glutathionylation of PTP1B, and PRDX oxidation using SDS-PAGE

Ligand stimulated cells were washed 1× with ice-cold phosphate-buffered saline (PBS; pH 7.4) and lysed with lysis buffer [0.5% Triton X-100, 0.5% sodium deoxycholate salt/deoxycholic acid, 150 mM NaCl, 20 mM tris (pH 7.5), 10 mM EDTA, and 30 mM sodium pyrophosphate (pH 7.5)]. The lysis buffer was supplemented with the PTP inhibitor sodium ortho-vanadate (200 μM) and a protease inhibitors cocktail (Roche). Protein concentrations in lysates were determined by Bradford assay and equal amounts of protein lysate was separated using SDS–polyacrylamide gel electrophoresis (PAGE). Proteins were then transferred to nitrocellulosa membranes (Millipore) and blocked with 5% milk in tris-buffered saline. Total phosphotyrosine 4G10 (Merck, no. 05-321) and the preferred site for PTP1B-mediated dephosphorylation site EGFR pY992 (Cell Signaling Technology) (1:1000) was determined by immunoblotting. Phosphotyrosine intensities was quantified and compared to total amount of loading as determined from EGFR intensities (R&D Systems, AF231). All the presented quantifications are derived from three independent biological replicates. To avoid underestimations caused by harsh stripping conditions, all the blots were performed on independent membranes. The samples for the three gels were prepared at the same time and run under identical conditions.

The oxidation state of PRDXs in A431 cells was determined by separation of the monomers and dimers forms by nonreducing SDS-PAGE (4 to 12% gel). Cells were washed once with PBS with subsequent blocking of reduced thiols through addition of 10 mM of *N*-ethylmaleimide in buffer [50 mM tris (pH 7.4), 150 mM NaCl, and catalase (1 μg/ml)] to the cells, as described previously (*38*). The cells were then washed and lysed with buffer containing 1% Nonidet P-40 (Sigma-Aldrich) and cOmplete protease inhibitors (Roche). Equal amount of cell lysates was resolved by SDS-PAGE and immunoblotted for PRDX1 (Cell Signaling Technology, 8499S), PRDX2 (Abcam, ab133481), and PRDX3 (AbFrontier, LF-MA0044). PRDX isoform dimer over total ratios were quantified using ImageJ software. Glutathionylation of recombinant PTP1B was determined by SDS-PAGE and immunoblotting using an antibody against GSH-protein complexes (ViroGen, 101-A).

### Detection of reversible oxidation of PTP1B in cells

The cysteinyl-labeling assay was performed to detect reversibly oxidized PTP1B in A431 cells, as previously described (*72*). The lysis buffer [50 mM sodium acetate (pH 5.5), 150 mM NaCl, 10% glycerol, 1% Surfact-Amp Nonidet P-40, aprotinin (5 μg/ml), leupeptin (5 μg/ml), and 10 mM iodoacetic acid (IAA)] was degassed, using a strong vacuum pump, and transferred into a hypoxic glove box equilibrated with 100% argon. Cells, cultured and treated as described above, were taken from a 37°C/5% CO_2_ environment to a 37°C incubator, treated with EGF ligand, and, at the end of incubation, rapidly transferred into the hypoxic glove box for subsequent handling under hypoxic conditions (<1% O_2_). At 4 min after EGF addition, the medium was immediately removed, and the cells were rapidly lysed with 800 μl of ice-cold degassed lysis buffer. The lysates were subsequently transferred to amber-colored microcentrifuge tubes, closing the lids under the hypoxic conditions. The tubes were subsequently taken out of the glove box and were shaken for 1 hour at room temperature to allow IAA-mediated alkylation of all accessible and thus reduced free thiol groups. Cleared lysates were subsequently applied to Zeba desalting spin columns to remove excess IAA. From each desalted sample, an aliquot for subsequent analysis of total PTP1B was taken off, after which the remaining sample was treated with 1 mM tris(2-carboxyethyl)phosphine (TCEP) for reduction of reversibly oxidized protein thiols. To label the thereby newly TCEP-reduced thiols with biotin, 5 mM EZ-link iodoacetyl-PEG2-biotin probe (Pierce) was added to the lysate. The biotinylated proteins, representing reversibly oxidized protein species in the original sample (for subsequent analysis of “oxPTP1B”), were isolated using streptavidin-Sepharose beads, washed with lysis buffer (pH 5.5), resuspended in 20 μl of 4× Laemmli sample buffer, and heated at 90°C for 90 s. The different samples were then resolved using separate SDS-PAGE analyses run in parallel (one for “total PTP1B” and one for oxPTP1B), subjected to immunoblots using anti-PTP1B antibodies.

### Proximity ligation assay

A431 epidermoid carcinoma cells or hPTP1B-reconstituted/PTP1B^−/−^ MEF cells were seeded onto coverslips (1 × 10^5^ cells per coverslip). Antibodies for the PLA assay were evaluated through a set of control experiments for interaction between PTP1B and GLRX1 (fig. S3A). After overnight starvation, the cells were stimulated with either EGF or PDGF (R&D Systems, catalog no. 220-BB) ligand for 4 min. Subsequently, RTK inhibitor [EGFRi (Sigma-Aldrich, no. 324674-1MG) or PDGFRi (Nintedanib; R&D Systems, no. 7049)] was added to block further phosphorylation. Cells were washed twice in PBS and fixed by using 4% paraformaldehyde for 10 min at room temperature. After three times wash in PBS, cells were permeabilized with 0.2% Triton X-100 in PBS for 15 min at room temperature. After permeabilization, cells were blocked with blocking buffer (Duolink PLA Reagents) in a preheated humidity chamber and placed in a 37°C incubator for 1 hour and then incubated at 4°C overnight with primary antibodies mouse anti-PTP1B (Sigma-Aldrich, FG6; 1:100 dilution), goat anti-GLRX1 (Biotech, AF3399-SP; 1:50 dilution), and rabbit hTXN1 (IMCO Corporation Ltd AB, 1:25 dilution) diluted in antibody diluent (Duolink PLA Reagents). Cells were washed three times with buffer A (Duolink PLA Reagents), followed by incubation for 1 hour at 37°C with secondary antibodies, Duolink In Situ PLA Probe Anti-Mouse MINUS, Anti-Rabbit PLUS, and Anti-Goat PLUS. PLAs were performed according to the manufacturer’s protocol (Olink Bioscience) (*39*, *40*). The slides were mounted by using Duolink In Situ Mounting Medium with 4′,6-diamidino-2-phenylindole. A Leica Dmi8 was applied for visualization and image acquisition. At least three random fields per slide were imaged and later quantified by ImageJ. The area of PLA signal per cell was counted, and the numbers were normalized relative to the control group.

### Statistical analyses

Data were analyzed using one-way analysis of variance followed by Bonferroni post hoc tests for multiple comparisons with Graph-Pad Prism.

## Supplementary Materials

### This PDF file includes:

Figs. S1 to S5 Uncropped Western blots
