# Supplementary material for: Intracellular enzymatic reducing systems control receptor tyrosine kinase signaling via PTP1B
Source: Sci Adv. 2026 Apr 10;12(15):eadv5362. doi: 10.1126/sciadv.adv5362 (PMC13068064; doi:10.1126/sciadv.adv5362)
Supplement: Supplementary file 1 — Figs. S1 to S5 Uncropped Western blots [file sciadv.adv5362_sm.pdf]

Science Advances
AAAS

Supplementary Materials for

Intracellular enzymatic reducing systems control receptor tyrosine kinase signaling via PTP1B

Lucia Coppo *et al.*

Corresponding author: Lucia Coppo, lucia.coppo@ki.se; Elias S. J. Arnér, elias.arner@ki.se; Markus Dagnell, markus.dagnell@ki.se

Sci. Adv. **12**, eadv5362 (2026)
DOI: 10.1126/sciadv.adv5362

This PDF file includes:

Figs. S1 to S5
Uncropped Western blots

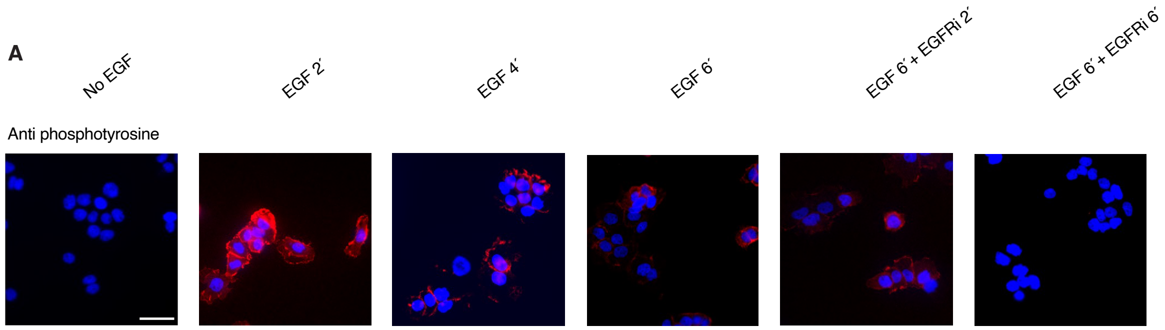

Fig. S1.

Fig. S1. ***Representative image of tyrosine phosphorylation staining following EGF stimulation in A431 cells.*** A431 cells were cultured on coverslips, serum-starved overnight, and stimulated with EGF in the presence or absence of an EGFR inhibitor for the indicated time points. Cells were then fixed with 4% paraformaldehyde and stained for phosphotyrosine (red) and nuclei (blue). Scale bar: 20 µm.

Fig. S2.

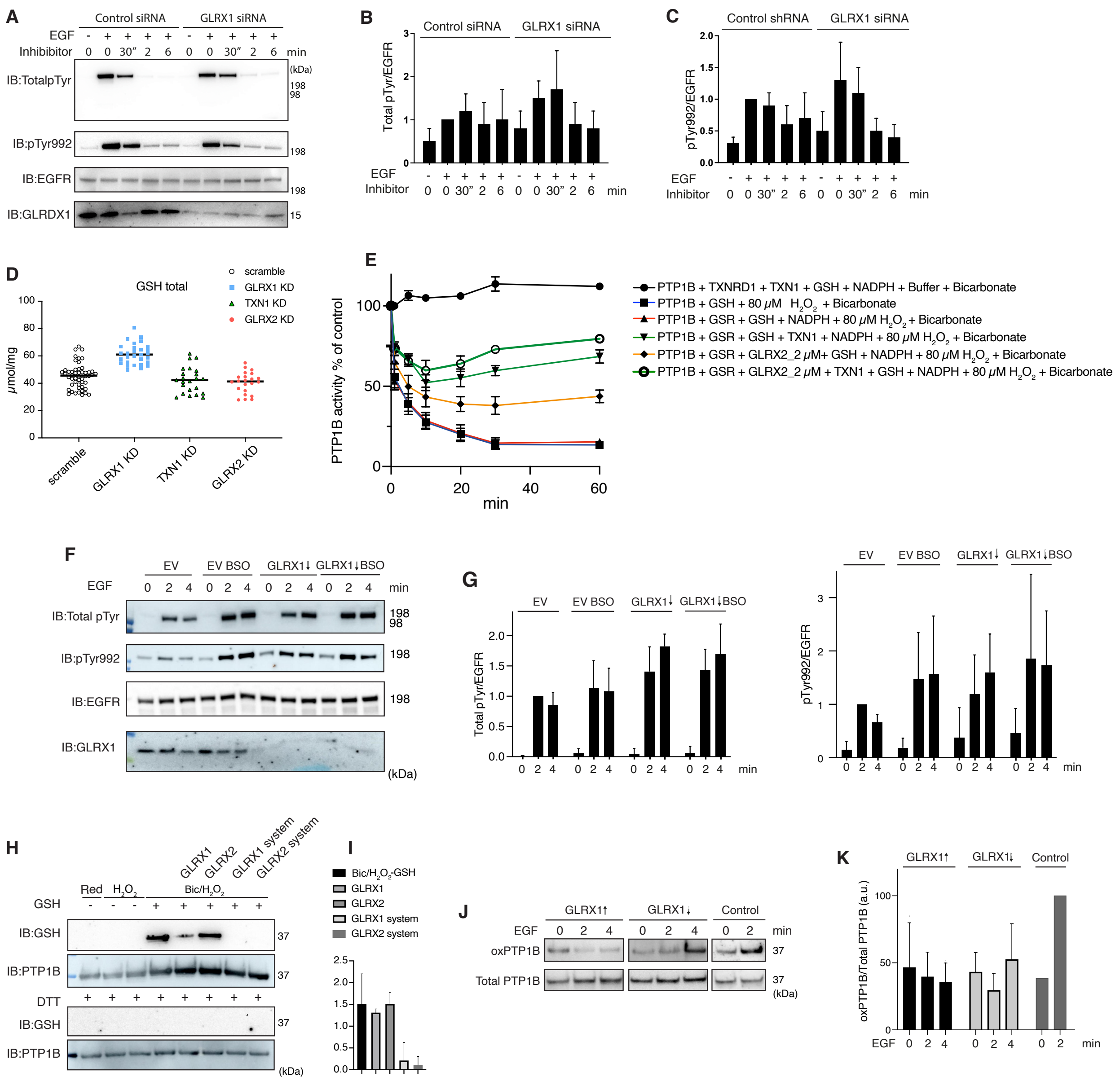

Fig. S2. ***Knockdown of GLRX1 leads to an increase in total GSH.*** (**A**), A431 cells with or without knockdown of GLRX1 were serum-starved before stimulation with EGF (100 ng/ml) for 4 min, after which EGFR receptor inhibitor (10 μM) was added. Cells were harvested at indicated times after addition of inhibitor, and receptor phosphorylation, (Tot pTyr and pY992) was analyzed by immunoblotting. The same samples were also used to confirm the knockdown of GLRX1. (**B** and **C**), The densitometric signals were normalized for the intensity of the total expression of the EGF-receptor (EGFR). Quantifications and densitometry are shown for (B), total pTyr and (C), pTyr992. (**D**), Analyses of total GSH in A431 cells subjected to GLRX1, GLRX2 and TXN1 siRNA. (**E**), Purified recombinant PTP1B was treated with H_2O_2 (80 μM) and bicarbonate (25 mM), in presence of 2 mM GSH and or GLRX2, 400 μM NADPH) and/or wild type TXN1 (10 μM). Measurements

of PTP activity was performed at the indicated times points (n=3 ± SEM). (**F** and **G**), A431 cells overexpressing GLRX1, indicated with upward arrows, were serum-starved before stimulation with EGF (100 ng/ml) for 0-2-4 min and analyzed as in A. Graphs are determinations from immunoblots of three separate experiments (n = 3; mean +/- SE (error bars); *, $p < 0.05$). (**H**), active recombinant PTP1B was treated with H_2O_2 (80 μM) with and without bicarbonate (25 mM) alone or in the presence of 2 mM GSH and enzyme components as indicated (2 μM GLRX1 or 2 μM GLRX 2) and NADPH, GSR). (**I**), The densitometric signals were normalized for the intensity of PTP1B-SSG signals over total amount of PTP1B. (**J** and **K**), A431 cells with or without knockdown of GLRX1 or overexpression of GLRX1 were serum-starved before stimulation with EGF (100 ng/ml). At the indicated times after EGF stimulation, cells were subjected to the modified cysteinyl-labeling assay using biotinylated iodoacetyl-PEG2-biotin for analysis of reversible PTP1B oxidation. Biotinylated proteins were purified using streptavidin-Sepharose beads and resolved by SDS-PAGE. Visualization was performed using antibodies against PTP1B and control levels were determined from total cell lysate by SDS-PAGE and blotting against PTP1B. The densitometric signals were normalized for the intensity of PTP1B streptavidin-biotin signals over total amount of PTP1B. Graphs are determinations from immunoblots of three (GLRX1) separate experiments (n = 3; mean +/- SE (error bars) *, $p < 0.05$). Data was analysed using one-way analysis of variance followed by Bonferroni post hoc tests for multiple comparisons with GraphPad Prism.

Fig. S3.

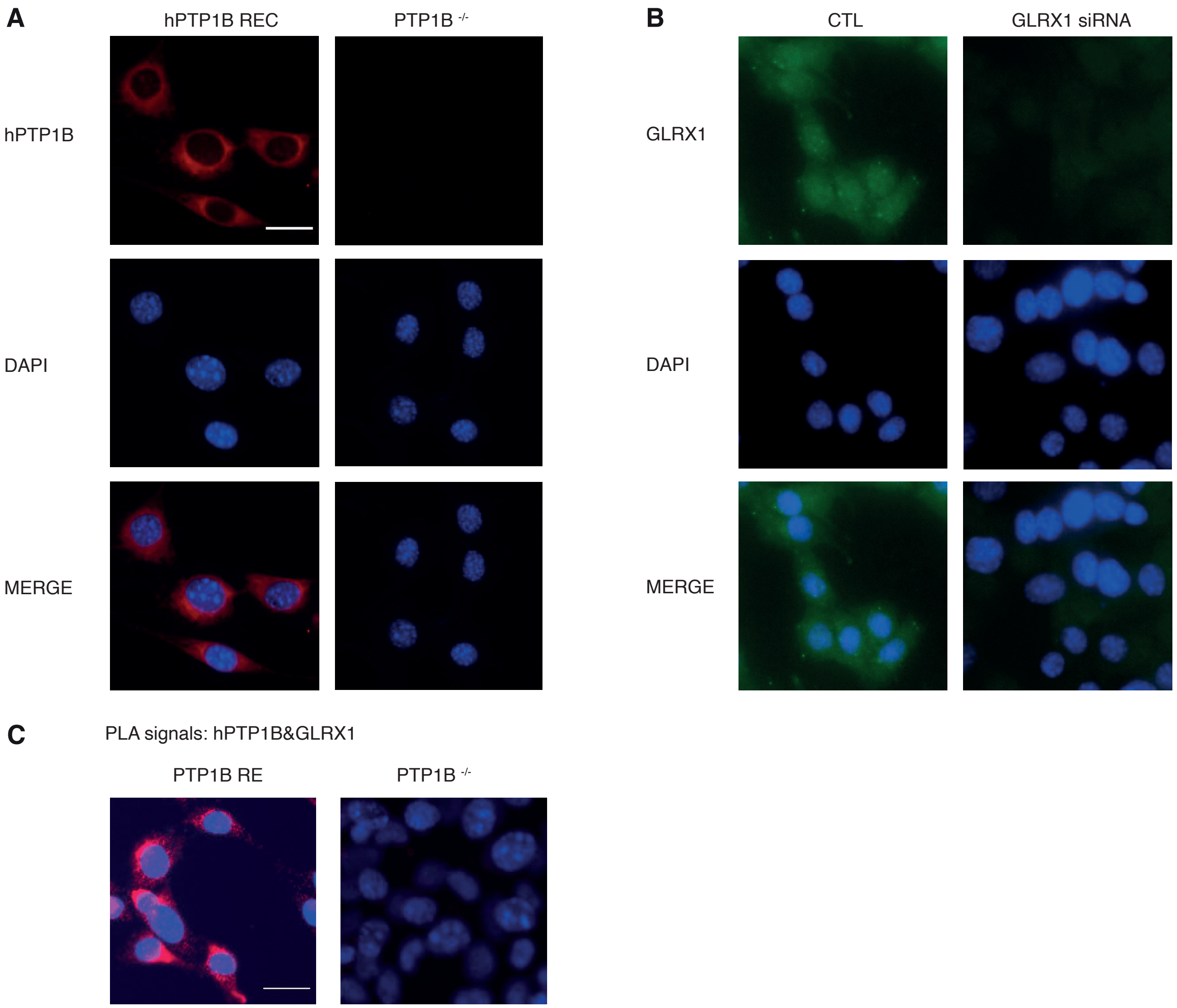

Fig. S3. ***Validation of GLRX1 and PTP1B Antibody Specificities for PLA assay in Knockdown Systems*** (**A**), PTP1B immunocytochemistry staining (red) in PTP1B-deficient (PTP1B$^{-/-}$) and hPTP1B-reconstituted MEF cells (hPTP1B) seeded overnight, fixed, permeabilized, and incubated with anti-hPTP1B primary antibody (mouse), followed by donkey anti-mouse Alexa 555 secondary antibody (1:1000). Scale bar: 30 µM. (**B**), GLRX1 immunocytochemistry staining (green) in hPTP1B-reconstituted MEF cells seeded overnight and transfected with siRNA targeting GLRX1 or with control siRNA, as indicated. Cells were fixed, permeabilized, and incubated with anti-GLRX1 primary goat antibody, followed by donkey anti-goat Alexa 488 secondary antibody (1:1000). Nuclei were stained with DAPI (blue). (**C**), PTP1B-deficient (PTP1B$^{-/-}$) and hPTP1B-reconstituted MEF cells (hPTP1B) were grown on coverslips, serum-starved and stimulated with 50 ng/ml PDGF for 4 min. Cells were subsequently fixed and permeabilized, whereupon a PLA was performed with anti-hPTP1B (mouse) and anti- GLRX1 (goat) primary antibodies, with the same protocol as in Fig 3. Nuclei were stained with DAPI (blue). Scale bar: 30 µM.

Fig. S4.

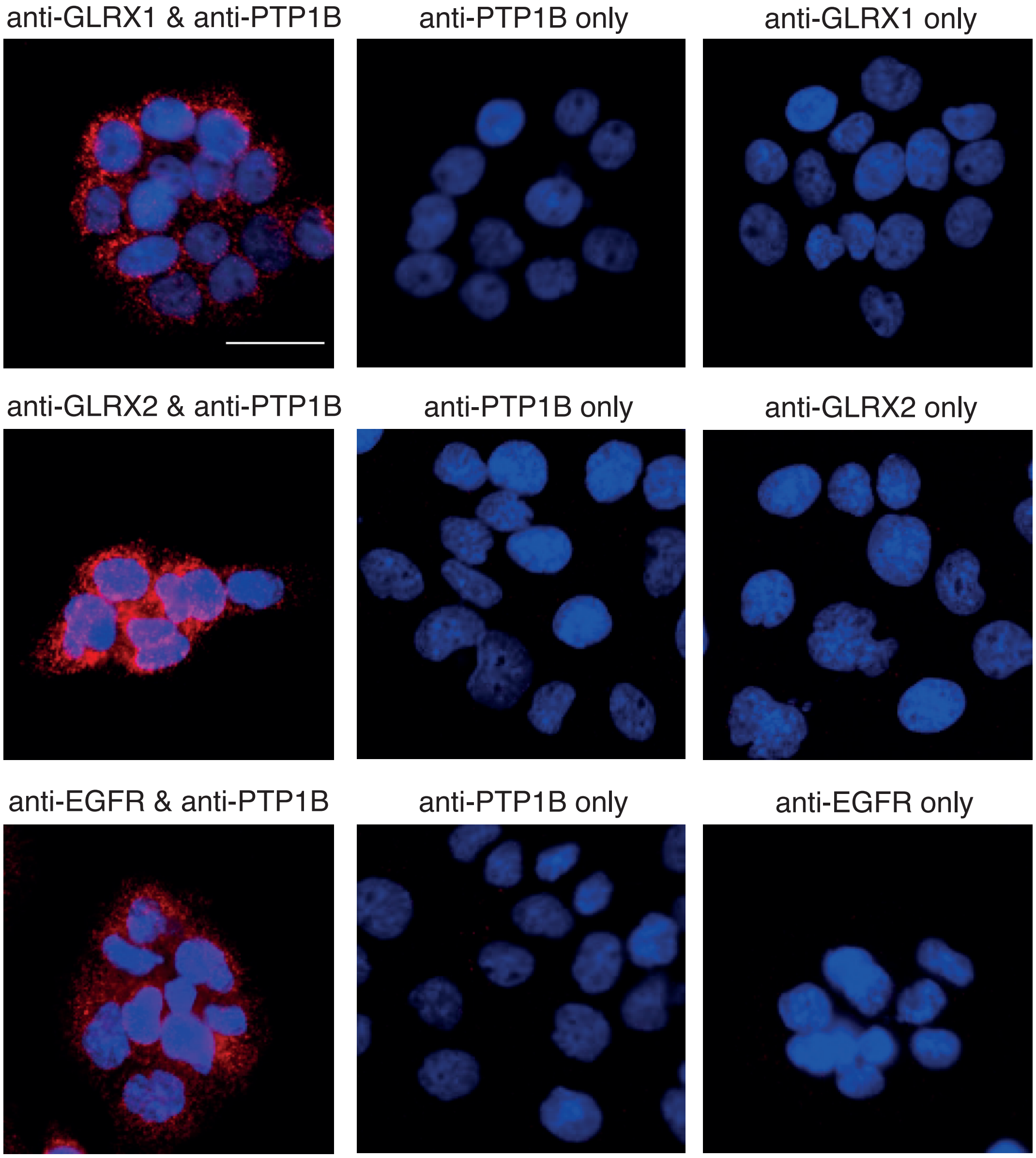

Fig. S4. ***Omission of One Primary Antibody in PLA Assay Demonstrates No Non-Specific Binding.*** A431 cells were cultured on coverslips overnight, fixed, permeabilized, and then stained with primary antibodies as indicated. PLA reactions were performed as described in Fig. 4 for detection of protein-protein interactions *in situ*. Negative controls were prepared by omitting one of the primary antibodies within each combination, as indicated. Fluorescence microscopy was used for visualization and red dots indicate positive PLA signals. Nuclei were stained with DAPI (blue). Scale bar: 30 μm.

Fig. S5.

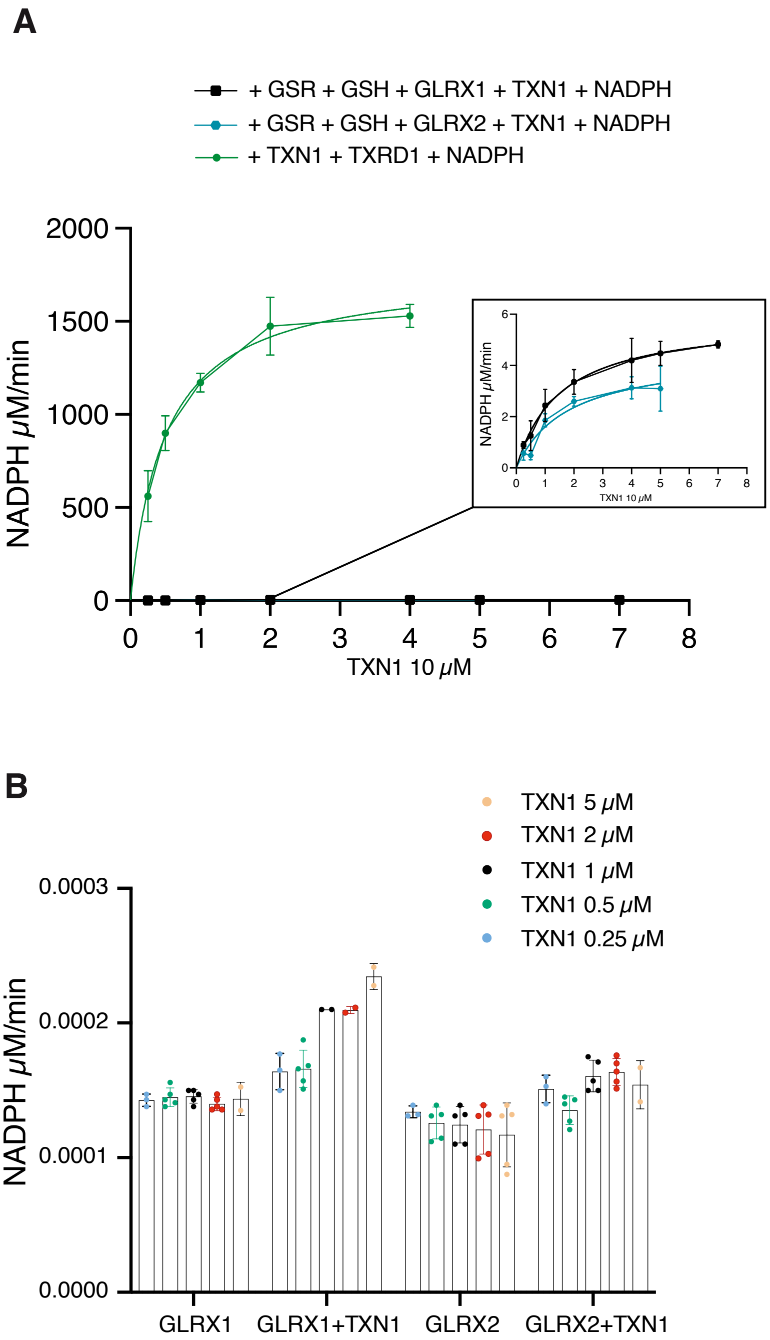

Fig. S5. ***TXN1 activity was measured using the insulin reduction assay.*** *(***A**), NADPH consumption was determined through the decrease of absorbance at 340 nm during the initial linear phases of the reactions with increasing concentrations of TXN1 (0 – 10 µM) in the presence of the GLRX1/2-GSH system (GSR (30 nM), GLRX1/2 (2 µM) and GSH (4 mM) or the complete Trx system (TXNRD1, TXN1 and NADPH), as indicated, with the inset showing activities in the absence of TrxR1. (**B**), Summary of the GLRX-system coupled data from (A) presented as a bar graph. (n=3 ± SD). Data was analysed using one-way analysis of variance followed by Bonferroni post hoc tests for multiple comparisons with GraphPad Prism.

Uncropped Western blots

Figure. 2.

5A1 4G10 (reverse order loading on gel)

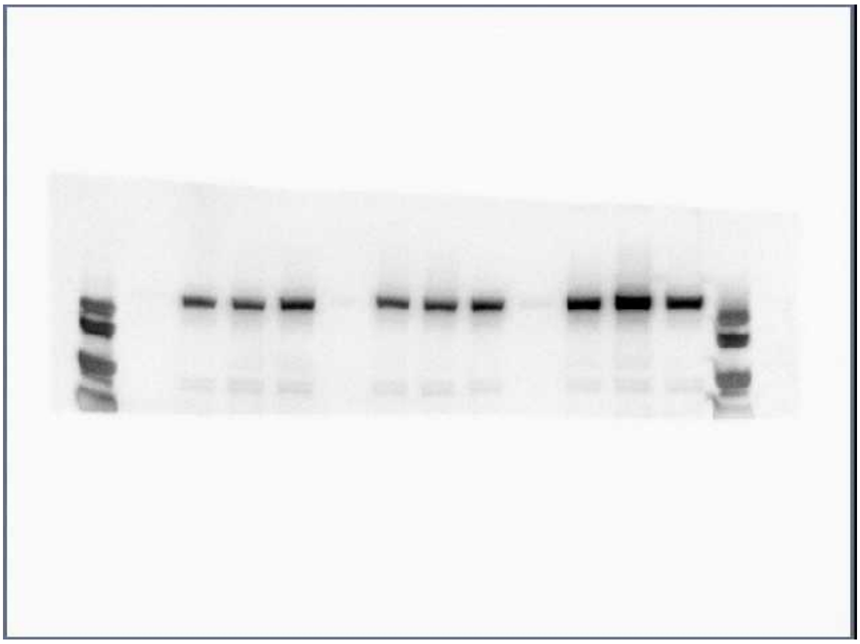

5A1 – p992

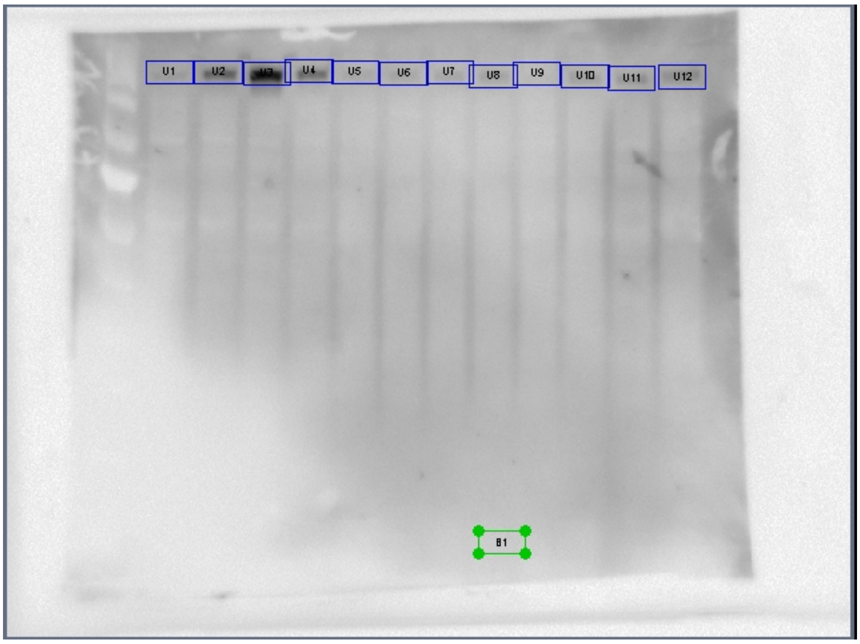

5A1 Total EGFR

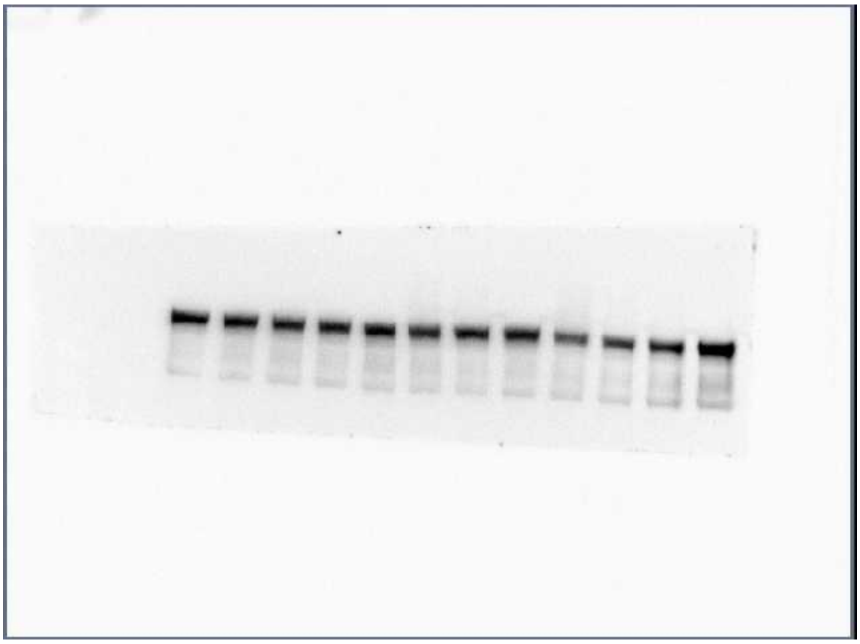

5A1 – Grx1 overexpression

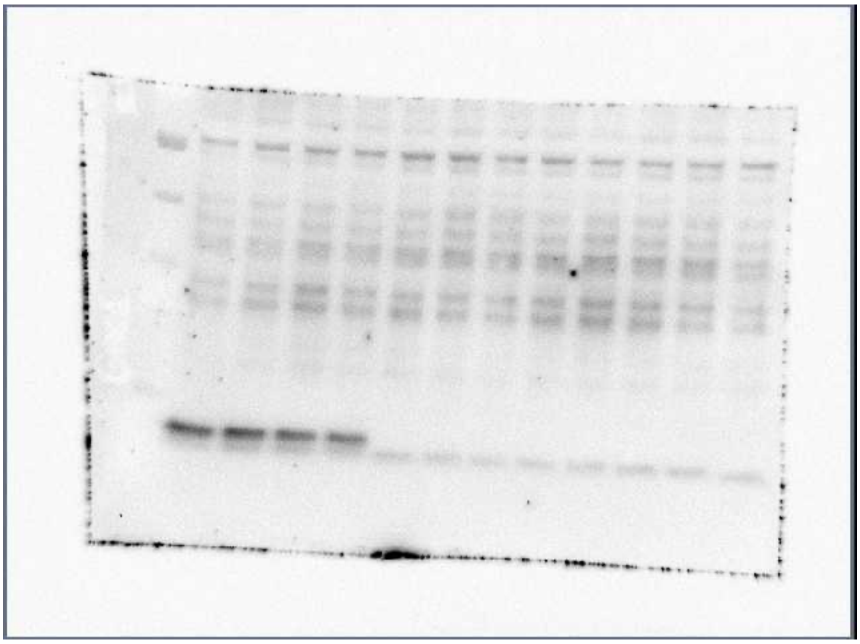

5A1 Grx2 overexpression

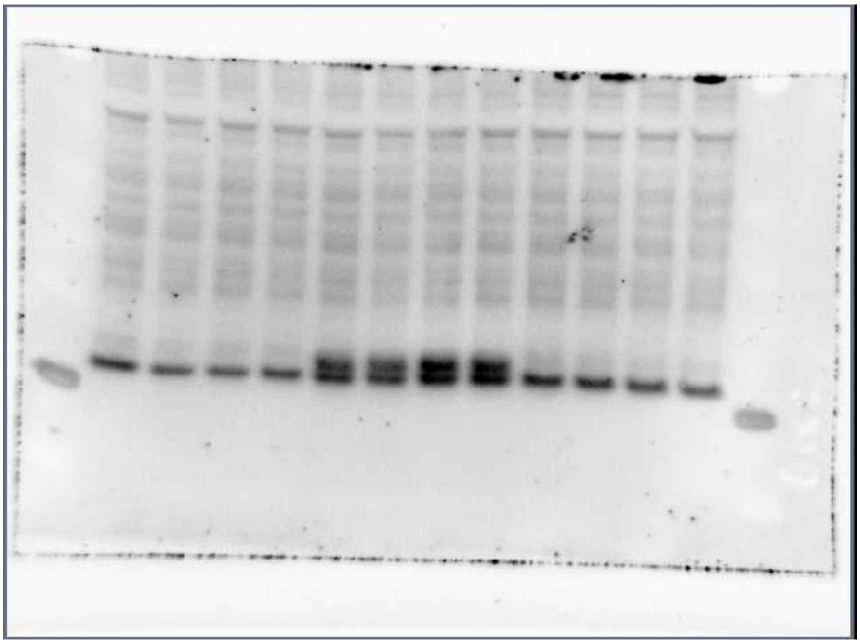

5A2 - 4G10 (total phosphorylation)

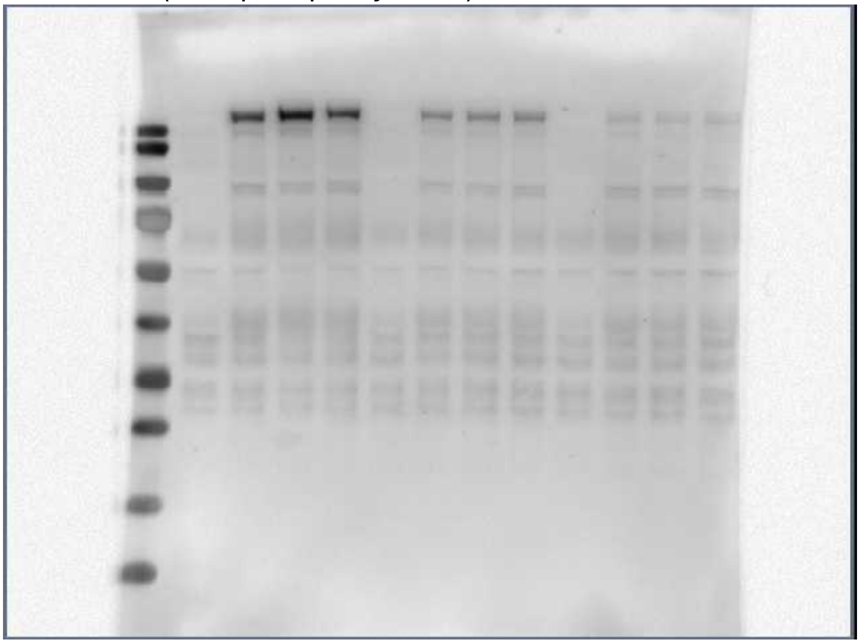

5A2 – p992

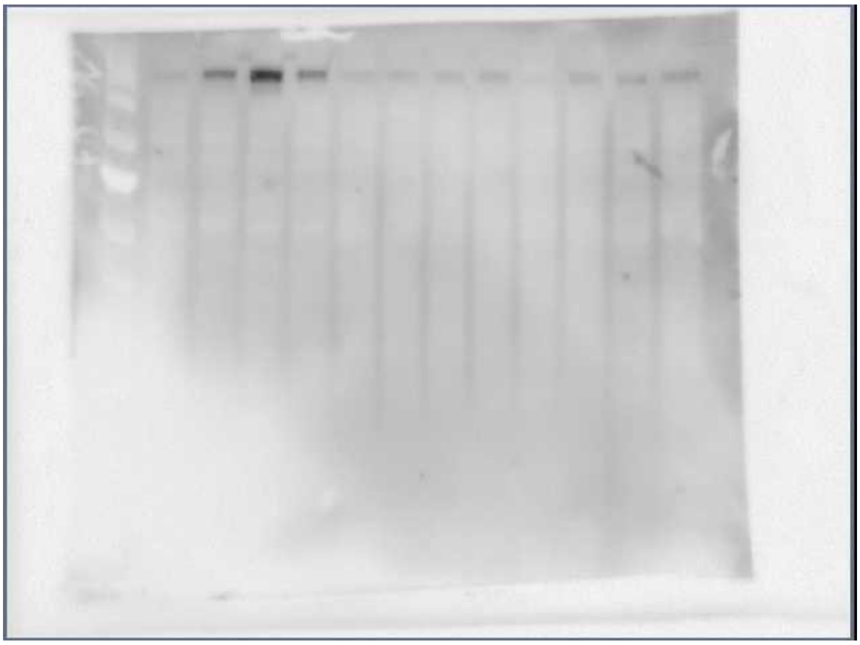

5A2 - Total EGFR

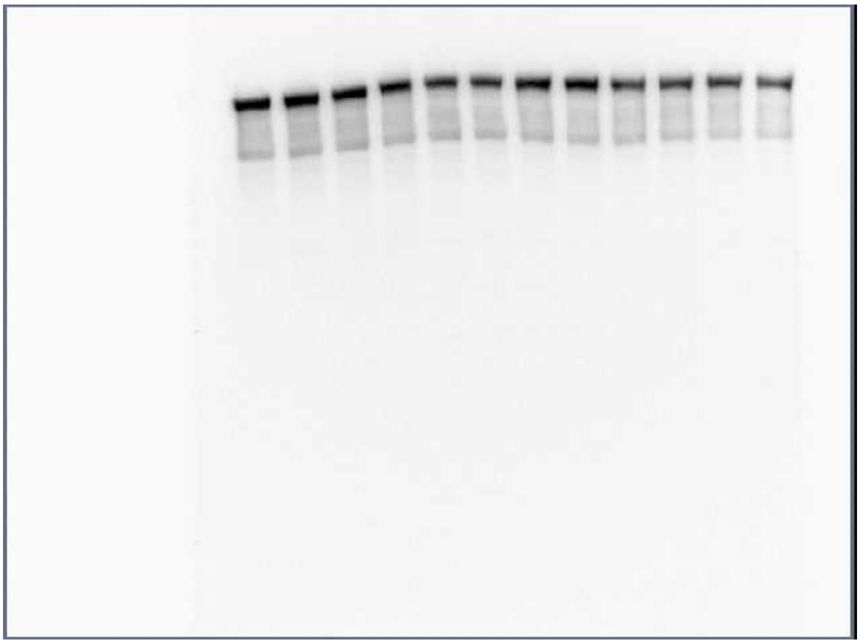

5A2 - Grx1 over expression

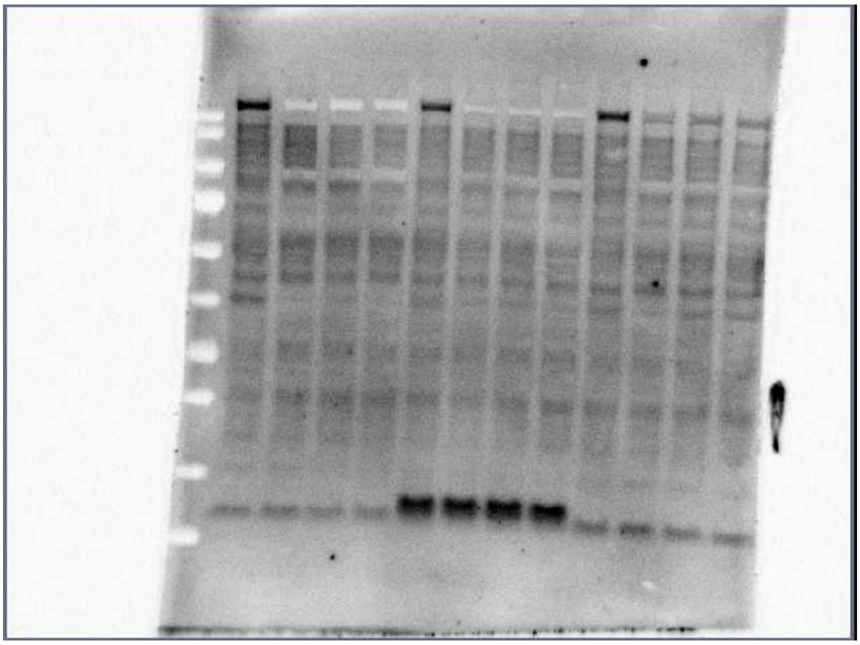

5A2 – Grx2 over expression

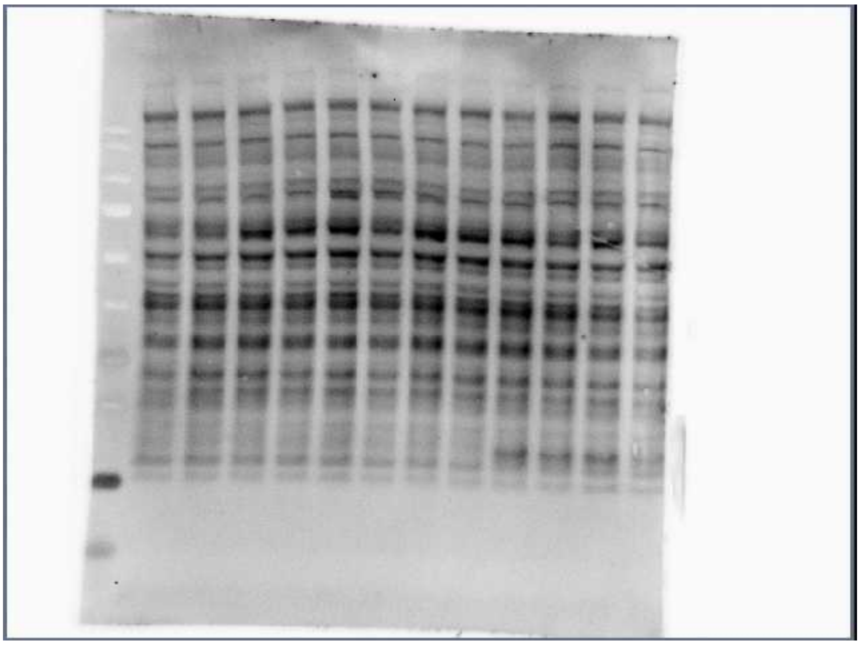

5A3

4G10 total phospho

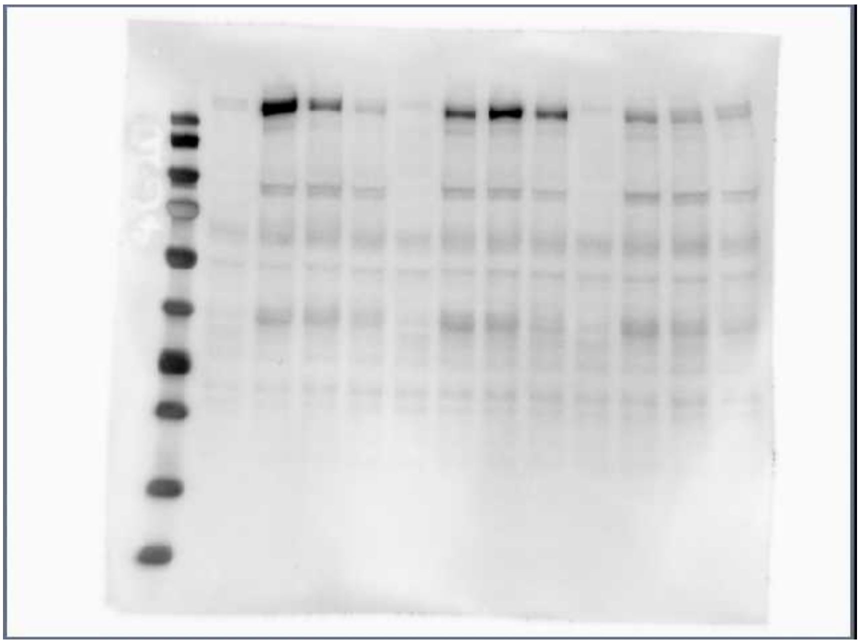

Total EGFR

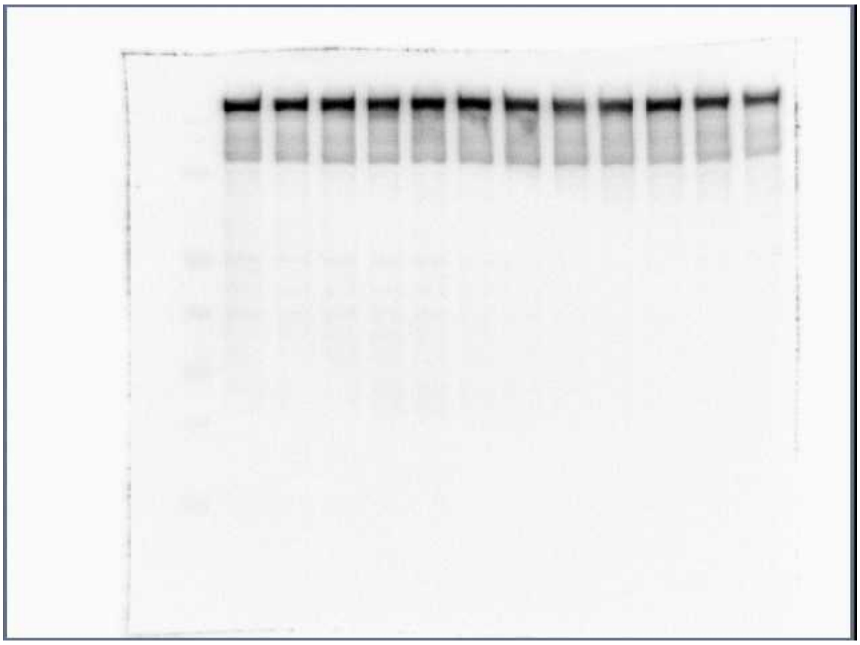

Grx1 overexpression

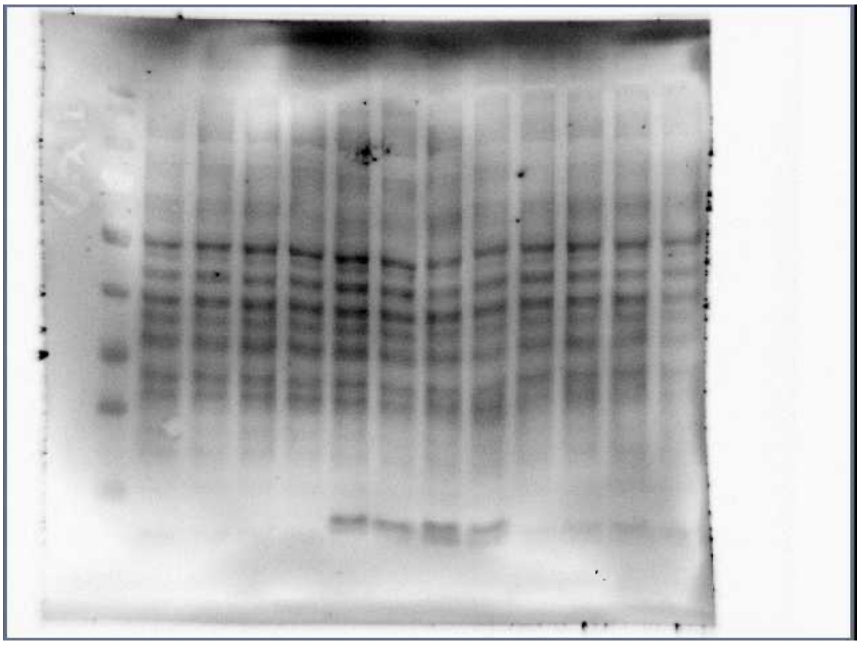

Grx1 overexpression

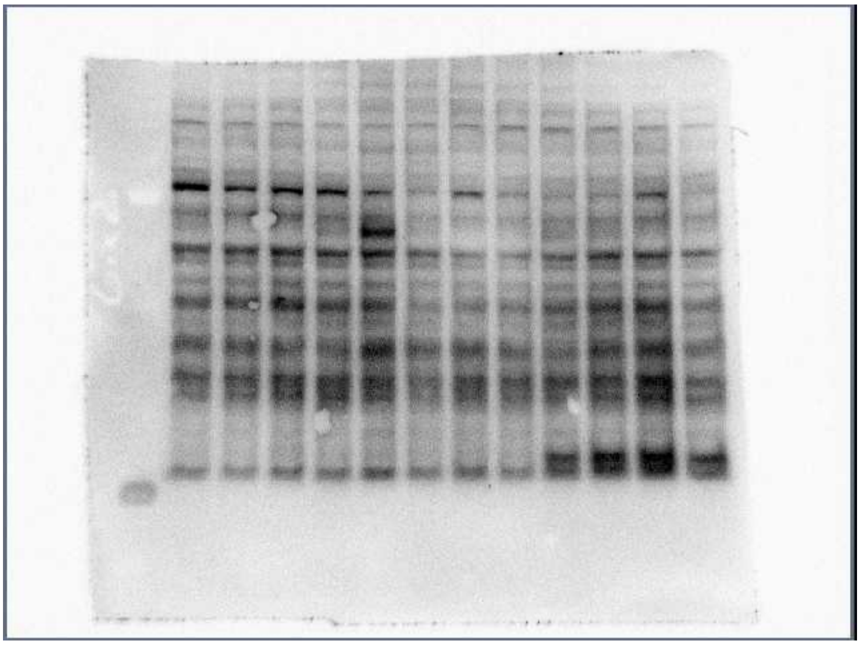

21-09-29 - 4G10 total phosphor

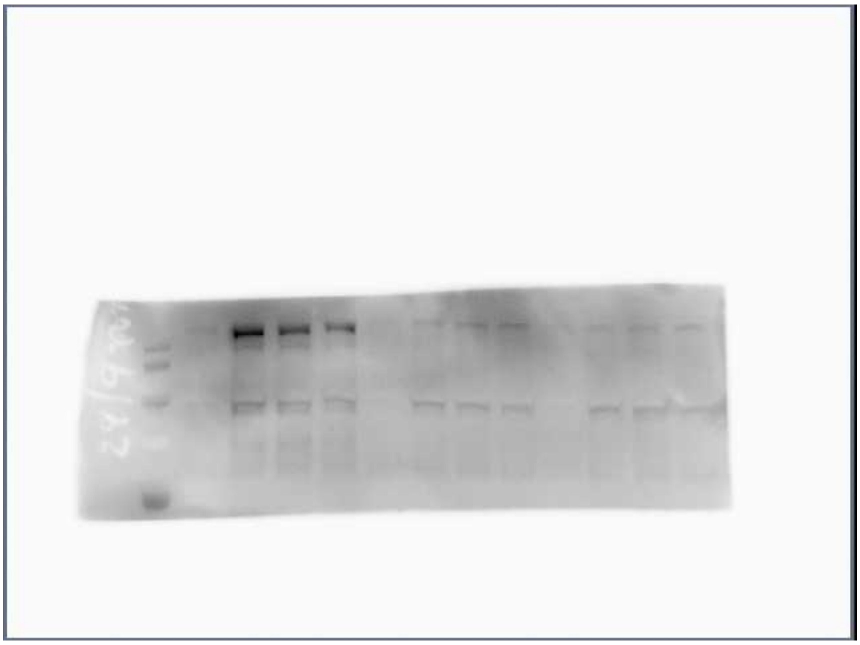

21-09-29 - Phospho 992

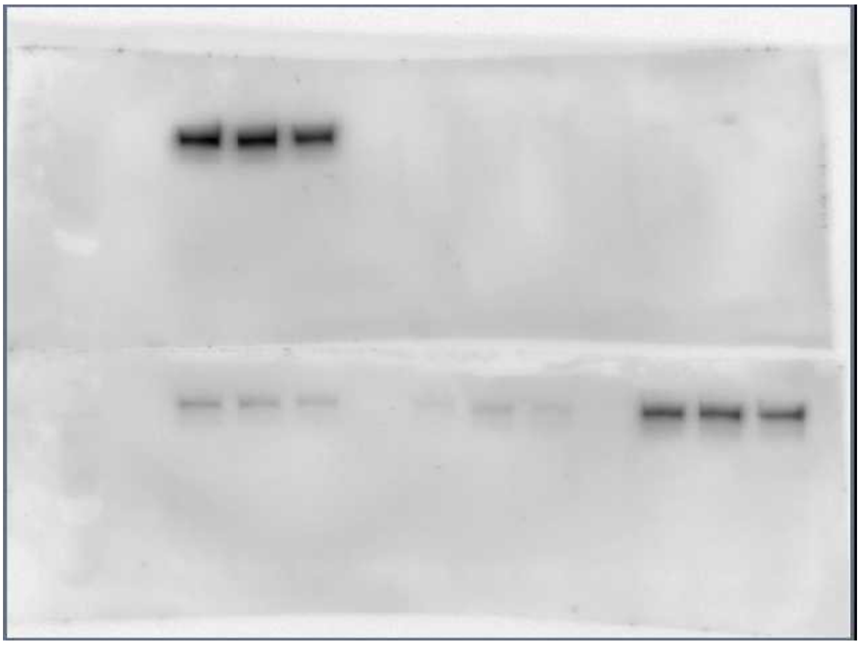

21-09-29 - Total EGFR

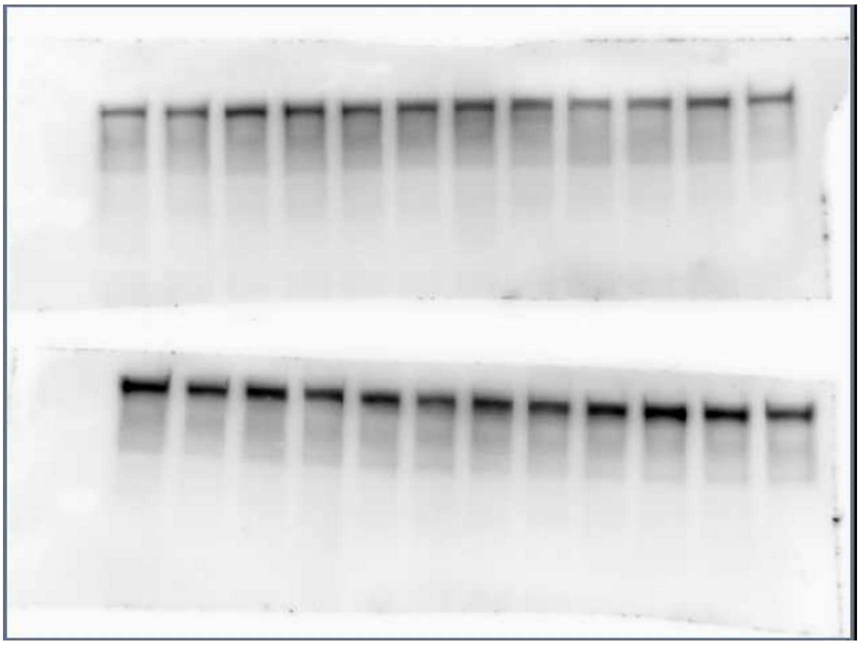

21-09-29 - Grx1 over expression

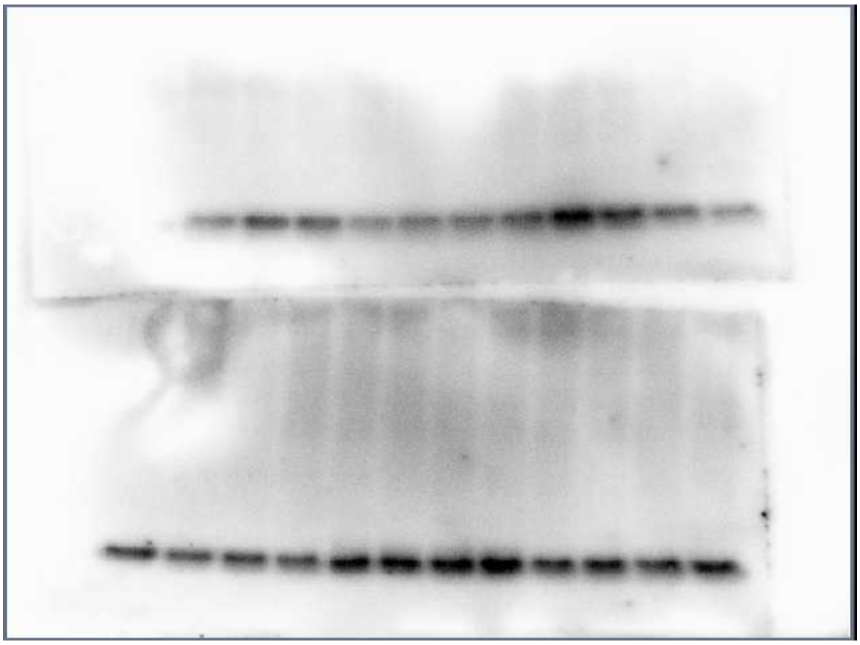

21-09-29 - Grx2 over expression

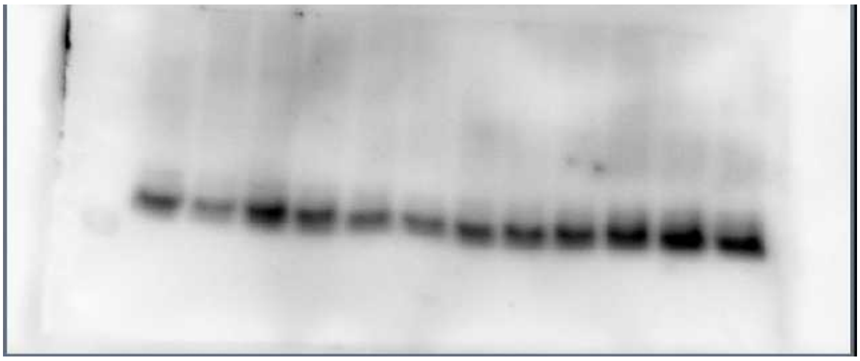

211001 - 4G10 Total phospho

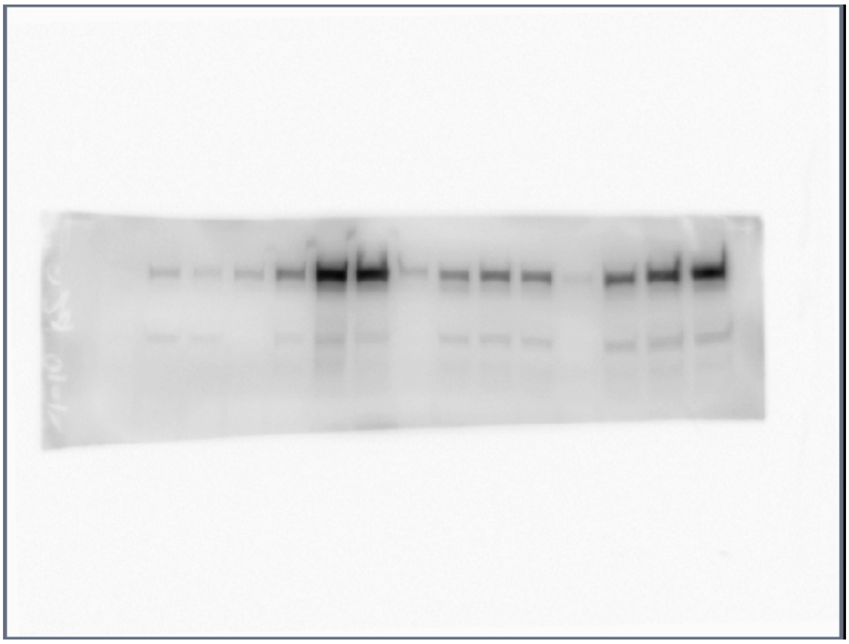

Supplementary Figure S2

Exp 2024-01-24

Sh 0--4’—4’+30”--4’+2’--4’+6’—Grx1KD

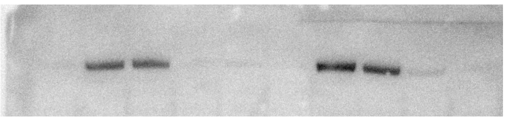

p-EGFR new gel doc

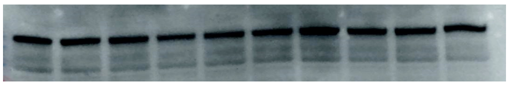

tot-EGFR new gel doc

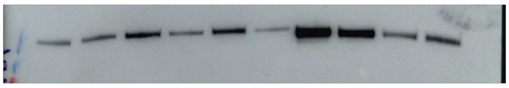

Site specific -EGFR new gel doc

Exp 2024-01-25

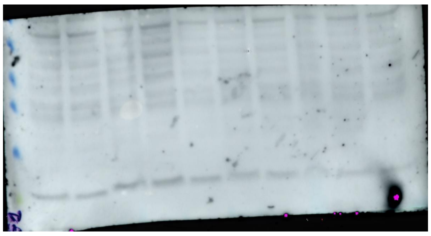

Grx1 -new gel doc

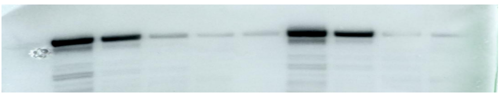

p-EGFR new gel doc

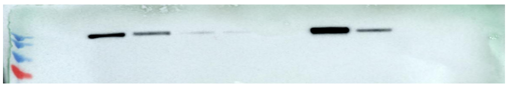

Site specific -EGFR new gel doc

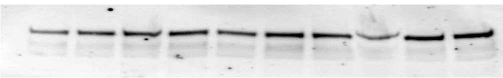

tot-EGFR new gel doc

Exp 2024-02-01

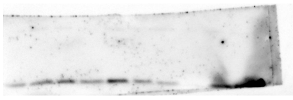

Grx1 -new gel doc

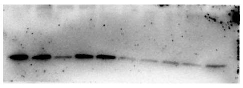

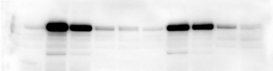

p-EGFR new gel doc

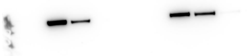

Site specific -EGFR new gel doc

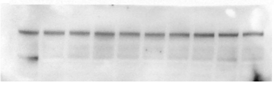

tot-EGFR new gel doc

Exp 2024-02-07

p-EGFR new gel doc

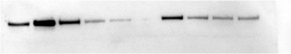

Site specific -EGFR new gel doc

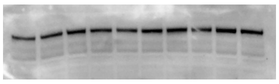

tot-EGFR new gel doc
